# Deletion of Serum Amyloid A3 Improves High Fat High Sucrose Diet-Induced Adipose Tissue Inflammation and Hyperlipidemia in Female Mice

**DOI:** 10.1371/journal.pone.0108564

**Published:** 2014-09-24

**Authors:** Laura J. den Hartigh, Shari Wang, Leela Goodspeed, Yilei Ding, Michelle Averill, Savitha Subramanian, Tomasz Wietecha, Kevin D. O'Brien, Alan Chait

**Affiliations:** 1 Division of Metabolism, Endocrinology, and Nutrition, Department of Medicine, University of Washington, Seattle, Washington, United States of America; 2 Division of Cardiology, Department of Medicine, University of Washington, Seattle, Washington, United States of America; Johns Hopkins University School of Medicine, United States of America

## Abstract

Serum amyloid A (SAA) increases in response to acute inflammatory stimuli and is modestly and chronically elevated in obesity. SAA3, an inducible form of SAA, is highly expressed in adipose tissue in obese mice where it promotes monocyte chemotaxis, providing a mechanism for the macrophage accumulation that occurs with adipose tissue expansion in obesity. Humans do not express functional SAA3 protein, but instead express SAA1 and SAA2 in hepatic as well as extrahepatic tissues, making it difficult to distinguish between liver and adipose tissue-specific SAA effects. SAA3 does not circulate in plasma, but may exert local effects that impact systemic inflammation. We tested the hypothesis that SAA3 contributes to chronic systemic inflammation and adipose tissue macrophage accumulation in obesity using mice deficient for *Saa3* (*Saa3* ^−/−^). Mice were rendered obese by feeding a pro-inflammatory high fat, high sucrose diet with added cholesterol (HFHSC). Both male and female *Saa3* ^−/−^ mice gained less weight on the HFHSC diet compared to *Saa3^+/+^* littermate controls, with no differences in body composition or resting metabolism. Female *Saa3* ^−/−^ mice, but not males, had reduced HFHSC diet-induced adipose tissue inflammation and macrophage content. Both male and female *Saa3* ^−/−^ mice had reduced liver *Saa1* and *Saa2* expression in association with reduced plasma SAA. Additionally, female *Saa3* ^−/−^ mice, but not males, showed improved plasma cholesterol, triglycerides, and lipoprotein profiles, with no changes in glucose metabolism. Taken together, these results suggest that the absence of *Saa3* attenuates liver-specific SAA (i.e., SAA1/2) secretion into plasma and blunts weight gain induced by an obesogenic diet. Furthermore, adipose tissue-specific inflammation and macrophage accumulation are attenuated in female *Saa3* ^−/−^ mice, suggesting a novel sexually dimorphic role for this protein. These results also suggest that *Saa3* influences liver-specific SAA1/2 expression, and that SAA3 could play a larger role in the acute phase response than previously thought.

## Introduction

Chronically elevated SAA associates with obesity and T2DM in humans and in mice [1]–[4]. Members of the serum amyloid A (SAA) family are acute phase proteins released in response to inflammation [5], and have been implicated in chronic inflammatory diseases such as rheumatoid arthritis [6], [7], atherosclerosis [8], [9], and obesity [2], [10]. There are four known human subtypes of SAA (SAA1–4), which vary in their alleged function and tissue expression. SAA1 and SAA2 are closely related subtypes primarily expressed by the liver. Circulating levels of SAA1 and SAA2 can rapidly increase up to 1000-fold in response to inflammatory stimuli such as lipopolysaccharide (LPS), silver nitrate, and casein, and are primarily associated with HDL in the blood [11]. Conversely, SAA3 is expressed in most extrahepatic tissues as well as the liver, with the highest expression found in adipocytes, lung, macrophages, and large intestine [12], [13]. SAA3 has not been found in blood using sensitive mass spectrometric techniques [14], [15] and therefore presumably performs paracrine/autocrine functions. Furthermore, while SAA3 is highly expressed in some mammals such as mice, it has evolved as a pseudogene in humans [16], where it is thought to be replaced functionally in obese adipose tissue by SAA1 and SAA2. SAA4 is expressed constitutively by most cells and responds only moderately to inflammatory stimuli [17].

Precise functions of SAA are not well defined, but may include major roles in host defense, such as extracellular matrix degradation and chemoattraction of inflammatory cells such as monocytes [18]–[20], as well as lipid-associated functions. During the acute phase response, SAA becomes the major HDL-associated protein, potentially altering apoA1-mediated reverse cholesterol transport [21]–[25]. SAA subtypes have been highly conserved through evolution [26], suggesting that they play a crucial role in the response to inflammation. While murine SAA1 and SAA2 are 96% homologous, SAA3 is only 63% and 65% homologous to SAA1 and SAA2, respectively [27]. Coupled with its differential tissue expression, this suggests that adipose tissue SAA3 could play an as yet undefined role in chronic inflammatory diseases that differs from hepatic acute phase SAA. Moreover, little is known about the consequences of chronic elevation of adipose tissue SAA3 in inflammatory conditions such as obesity.

A characteristic feature of obesity is the accumulation of macrophages within adipose tissue [28]. We and others have shown that *Saa3* can be highly expressed by cultured adipocytes in response to inflammatory stimuli such as saturated fatty acids and 25 mM glucose [29], [30]. A primary function of adipocyte SAA3 appears to be chemoattraction of monocytes, similarly to monocyte chemotactic protein-1 (MCP-1, encoded by *Ccl2*) [31]. Furthermore, we have also previously shown that a high fat, high sucrose diet with and without added cholesterol not only induces obesity and systemic inflammation, but also increases adipose tissue *Saa3* expression and macrophage content [4], [29], [32]. Mice deficient in the MCP-1 receptor show diminished macrophage content within adipose tissue when placed on a high fat diet [33], although some macrophages still remain which suggests that other chemotactic factors for macrophage recruitment, such as SAA3, also might play a role.

Because humans do not express SAA3, a murine model specifically deleting SAA3 provides a unique opportunity to tease apart the effects of adipose tissue-specific SAA from hepatic SAA that would otherwise be difficult to do in humans. We hypothesized that the absence of SAA3 would reduce obesity-induced macrophage accumulation in adipose tissue, which could improve systemic inflammation. To test this hypothesis, we generated mice with specific global deletion of *Saa3*. *Saa3^−/−^* mice had restricted weight gain on an obesogenic diet. Female *Saa3^−/−^* mice also had reduced adipose tissue inflammation and macrophage content, and an improved systemic lipid profile. Liver-derived SAA production was also decreased in *Saa3^−/−^* mice, suggesting that SAA3 could play a significant role in hepatic inflammation and lipoprotein metabolism.

## Materials and Methods

### Generation of *Saa3^−/−^* mice

Targeted embryonic stem cells lacking the entire protein coding sequence of *Saa3* were derived from C57BL/6N mice by Regeneron Pharmaceuticals through the Knock Out Mouse Project (KOMP) at the University of California, Davis. Specific information on how these *Saa3*-null embryonic stems cells were generated can be found at http://www.velocigene.com/komp/detail/13807. Chimeric offspring were generated by injecting *Saa3*-null embryonic stems cells into albino C57BL/6 embryos. Germline transmission was confirmed by breeding male chimeras to albino females, yielding black pups that were heterozygous for the desired *Saa3* deletion. Wild type (*Saa3* ^+/+^, WT), heterozygous (*Saa3* ^+/−^, HET), and knock out (*Saa3* ^−/−^, KO) mice used in this study were obtained by breeding F2 generation *Saa3* ^+/−^ mice to yield littermates with all three genotypes represented. Mice from all three genotypes appeared normal with no obvious phenotype. A minimum of 6 mice were used in each treatment group. For simplicity, only results from the Saa3^+/+^ and *Saa3* ^−/−^ mice will be shown.

### Mouse study design

Ten-week-old adult male and female *Saa3* ^+/+^, *Saa3* ^+/−^, and *Saa3* ^−/−^mice were fed either normal chow (12% calories from fat) or a high fat, high-sucrose diet with 0.15% added cholesterol (HFHSC, F4997, Bioserv) ad libitum for 16 weeks. Details of these diets have been published previously [32]. The HFHSC diet provides 20.5% of calories as protein, 36% as fat (40% w/w saturated, 50% monounsaturated, and 10% polyunsaturated fats) and 36% as carbohydrate. The HFHSC diet was used in this study as we have previously shown that it induces significant *Saa3* gene expression in adipose tissue [32]. Body weights were measured weekly. At sacrifice, harvested tissues were snap-frozen in liquid nitrogen and stored at −70°C or were fixed with 10% neutral-buffered formalin and embedded in paraffin wax. All experimental procedures were undertaken with approval from the Institution Animal Care and Use Committee of the University of Washington. Terminal procedures were performed under isoflurane anesthesia, and every effort was made to minimize animal suffering.

### Quantitative real-time PCR

Total RNA was extracted from ∼100 mg of whole adipose or liver tissues using a commercially available RNA extraction kit according to the manufacturer's protocol (Qiagen RNeasy Mini Kit). After spectroscopic quantification, 2 µg of RNA was reverse-transcribed, and the cDNA thus obtained was analyzed by real-time quantitative PCR by standard protocols using an ABI 7900HT instrument. Primer and probe sets for individual genes were purchased from Applied Biosystems (Assay-on-Demand, Life Technologies, Carlsbad, CA). GAPDH was used as a housekeeping gene, levels of which did not change with the various diets or genotypes. Relative amounts of the target gene were calculated using the ΔΔCt formula and expressed as a fold change from the *Saa3* ^+/+^ chow animals.

### Body composition

Measurements of body lean and fat mass were determined in live, conscious animals by use of quantitative magnetic resonance spectroscopy (QMR; EchoMRI-700TM; Echo MRI) after 11 weeks on diet by the University of Washington Nutrition Obesity Research Center (NORC) Energy Balance and Glucose Metabolism (EBGM) Core.

### Indirect calorimetry and ambulatory activity

Mice were acclimated to calorimetry cages prior to the study and data collection. Energy expenditure measurements were obtained as described previously [34]. Briefly, measurements were collected using a computer-controlled open-circuit indirect calorimeter using the Oxymax Laboratory Animal Monitoring System (Columbus Instruments, Columbus, OH) with support from the EBGM Core of the NORC at the University of Washington, as previously described [35]. Oxygen consumption (V_2_) and carbon dioxide production (Vc_2_) were measured for each mouse every 20 minutes for 2-minute intervals, and food and water intake were measured using the feed-scale and volumetric drinking monitoring systems, respectively (Columbus Instruments). V_2_ was converted to total energy expenditure in kilocalories per hour by Columbus software, which uses the standard Lusk formula [TEE (kcal/h)  = (3.815+1.232× RQ) × V_2_ (l/h)], where respiratory quotient (RQ) is the ratio of V_2_ to Vc_2_ [36]. Locomotor activity was evaluated using an Opto-Varimetrix-3 sensor system (Columbus Instruments). Consecutive adjacent infrared beam breaks in the x-axis were scored as an activity count, and a tally was recorded every 20 min. Mice were evaluated over two consecutive 24-h periods, and we report the results for the dark (active) photoperiods.

### Plasma analyses

Lipoprotein classes were separated from pooled plasma samples by fast-phase liquid chromatography (FPLC). Triglycerides and cholesterol were measured from fasting plasma and pooled plasma FPLC fractions using colorimetric assays as previously described [3], and plasma insulin was measured using a commercially available kit, as described previously [37]. Plasma SAA was measured by ELISA [3].

### Liver lipids and histology

Lipids were extracted using the Folch technique as described previously [38]. Triglycerides and cholesterol were then measured using commercially available kits as described previously [39].

For histological analysis, formalin-fixed livers embedded in paraffin wax were sectioned at 4 µm thickness and stained with Trichrome Stain Masson Kit (Sigma-Aldrich). All stained tissue sections were visualized by Olympus BX50 microscope and then photographed using a Canon EOS 5D Mark II DSLR camera at 10X magnification. Images were analyzed using Image Pro Plus 6.0 (Media Cybernetics).

### Glucose homeostasis

Blood glucose was measured from each mouse every 4 weeks. Glucose tolerance testing was performed on fasted mice (4 hour fast) after 14 weeks on HFHSC diet. An intraperitoneal glucose dose of 1.5 mg/kg was administered followed by blood glucose measurements at 0, 15, 30, 60 and 120 minutes post injection from a tail nick. Insulin tolerance was determined on 4 hour-fasted mice after 15 weeks on HFHSC by injecting 1 mU/g human insulin intraperitoneally and measuring blood glucose at 0, 30, 60, and 120 minutes post-injection.

### Immunohistochemistry and adipocyte sizing

Formalin-fixed, paraffin-embedded adipose tissue was sectioned and stained with a rat monoclonal Mac2 antibody (1:2500 dilution, Cedarlane Laboratories) for relative quantification of adipose tissue macrophages as described previously [32]. Area quantification for Mac2 staining was performed on digital images of immunostained tissue sections using image analysis software (Image Pro Plus software, Media Cybernetics). To estimate mean adipocyte size, sections of gonadal adipose tissue were stained with Movat's Pentacrhome. One randomized photomicrograph was analyzed at 20X objective (Canon EOS 5D Mark II, Olympus BX50) and quantified by manually outlining each individual adipocyte by hand (Image Pro Plus/Media Cybernectics software, Wacom Cintiq 21UX tablet).

### Statistics

Data were analyzed using GraphPad Prism 6 software and are represented as means +/−standard errors. One- and two-way ANOVA (ANOVA) were used to compare differences between mice of different genotypes receiving the different diets as indicated, and Bonferroni post-hoc testing was used to detect differences among mean values of the groups. A *P* value <0.05 was considered statistically significant.

## Results

### Absence of *Saa3* blunts HFHSC-induced weight gain

**Figure 1:**
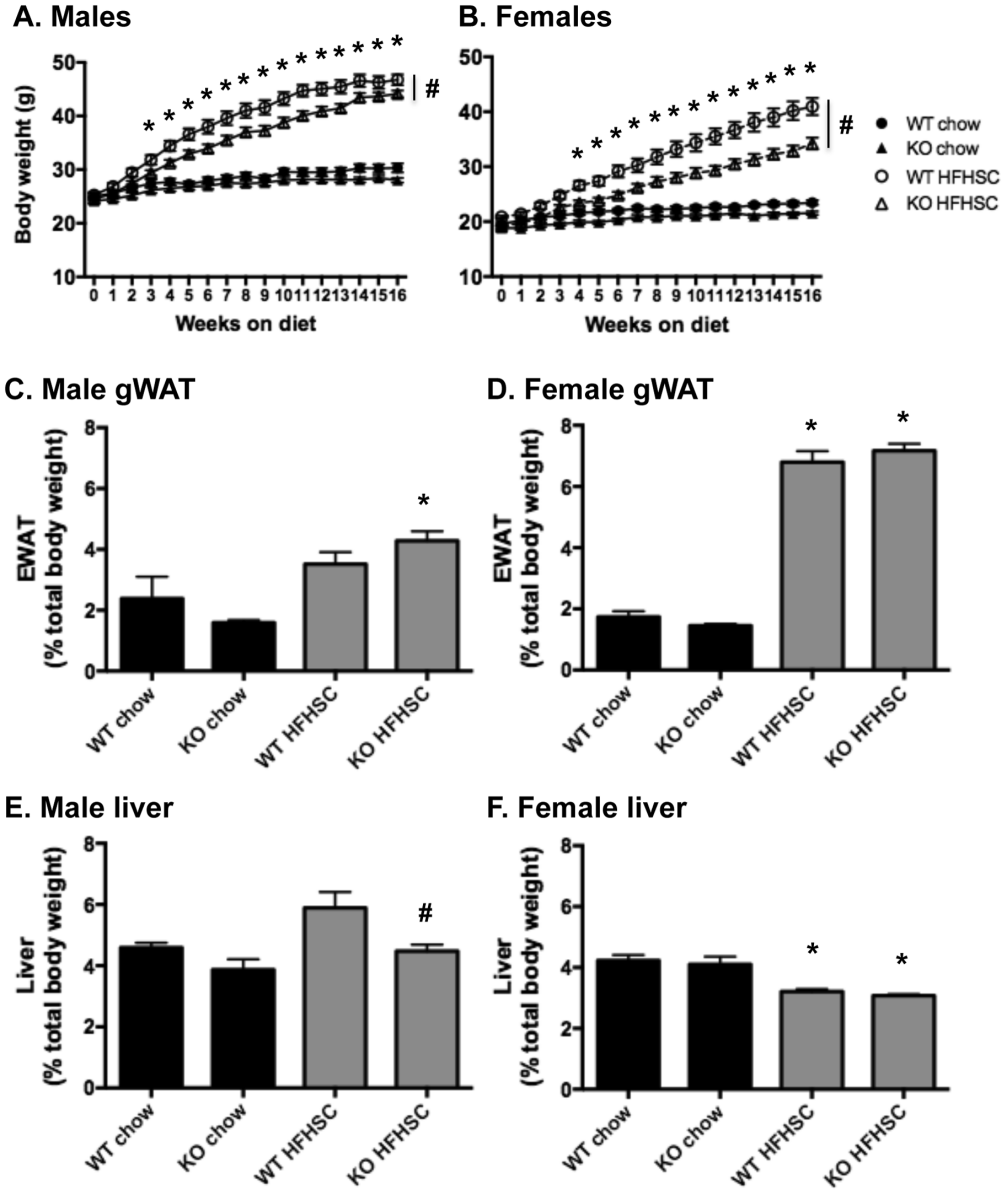
Deletion of *Saa3* attenuates weight gain on a HFHSC diet. (A) Male and (B) female *Saa3* ^+/+^ and *Saa3* ^−/−^ mice were fed chow or a high fat, high sucrose + cholesterol (HFHSC) diet for 16 weeks, and weekly body weights recorded. (C–D) Gonadal white adipose tissue (gWAT) and (E–F) liver weight were recorded at sacrifice and normalized to total body weight. n = 6–15 mice per group. *P<0.05 from chow group; #P<0.05 from *Saa3* ^+/+^ controls. WT: *Saa3* ^+/+;^ KO: *Saa3* ^−/−^.

*Saa3^+/+^* and *Saa3* ^−/−^ mice did not appear phenotypically different on a chow diet. However, on a HFHSC diet, both male and female *Saa3* ^−/−^ mice gained significantly less weight than their *Saa3* ^+/+^ counterparts, a trend that persisted over the 16-week dietary intervention (Figure 1A–B). At baseline male and female *Saa3^+/+^* mice weighed an average of 25.3 and 20.9 g, respectively, and after 16 weeks of HFHSC diet weighed 47.0 and 41.0 g. Conversely, male and female *Saa3^−/−^* mice began at 24.5 and 19.1 g, respectively, and after 16 weeks on the HFHSC diet weighed 43.2 and 34.2 g. Compared to *Saa3* ^+/+^ controls, male and female *Saa3^−/−^* mice gained 8.1% and 16.4% less weight, respectively. Despite significant differences in body weight, there were no differences in body composition relative to body weight between *Saa3* ^+/+^ and *Saa3* ^−/−^ mice after 11 weeks on chow or HFHSC diet (Figure S1 in File S1). Moreover, gWAT mass, expressed as a total percentage of body weight, did not differ between *Saa3* ^+/+^ and *Saa3* ^−/−^ male or female mice at sacrifice (Figure 1C–D). Liver mass was significantly decreased from 2.9 to 2.0 g in *Saa3* ^−/−^ male mice (Figure 1E, presented as a percent of total body weight), but not in female *Saa3* ^−/−^ mice (1.3 to 1.1 g, Figure 1F) compared to *Saa3* ^+/+^ HFHSC-fed controls. To explore additional mechanisms for the blunted weight gain in *Saa3* ^−/−^ female mice on HFHSC diet, we measured indirect calorimetry and resting activity. Female mice fed a HFHSC diet had higher V_2_, Vc_2_, and heat production, with a decrease in respiratory quotient (RQ) and food intake (Figure S2 in File S1). There were no differences in V_2_, Vc_2_, RQ, heat production, or physical activity between female *Saa3^−/−^* and *Saa3* ^+/+^ mice (Figure S2 in File S1). Furthermore, gWAT adipocyte size in female mice of different genotypes were not different, but male adipocytes were larger in *Saa3^−/−^* mice on a HFHSC diet (Figure S3 in File S1).

### Plasma lipids, but not glucose metabolism, are improved by deletion of *Saa3* in obese female mice

**Figure 2:**
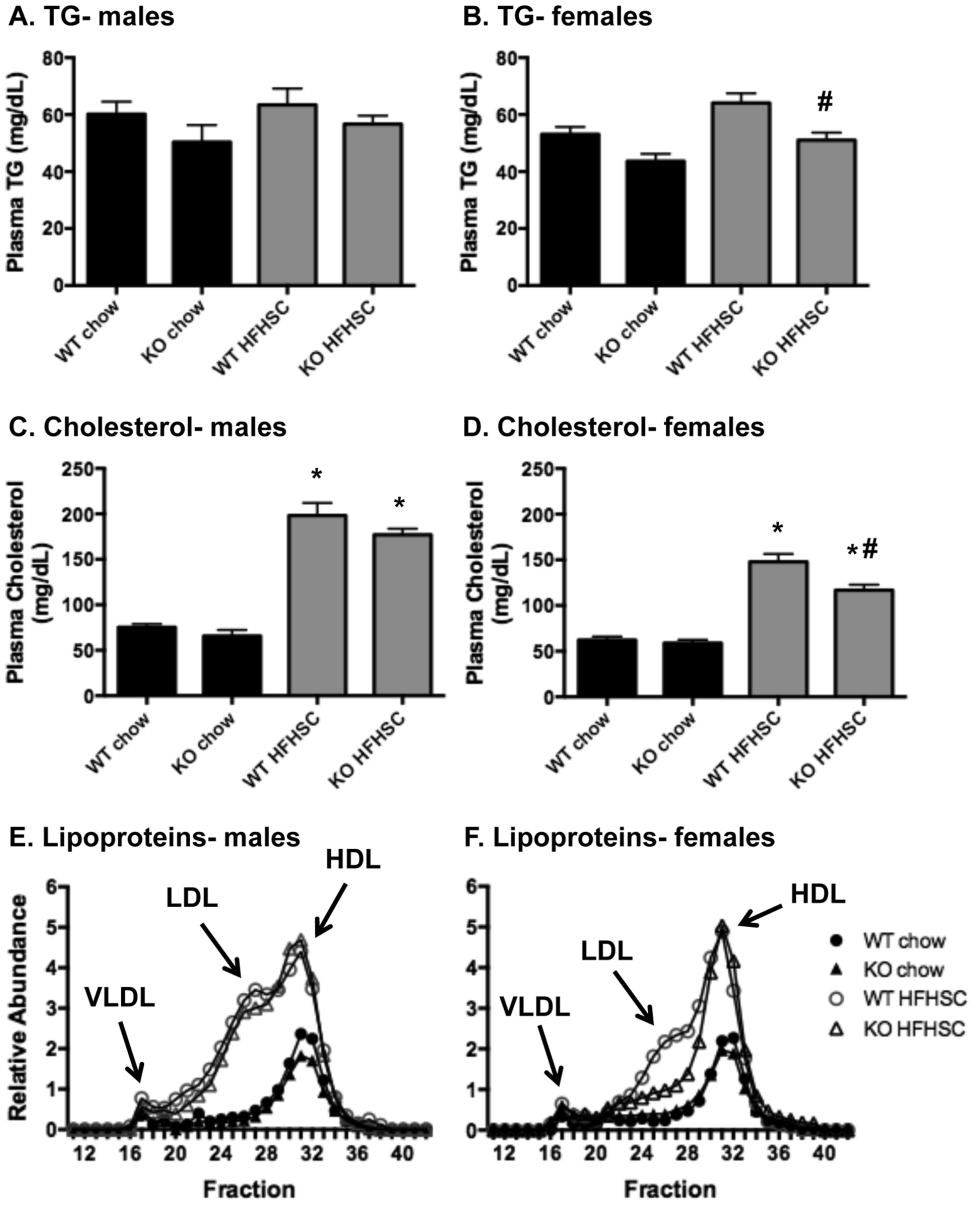
Plasma triglycerides, cholesterol, and lipoprotein profiles are improved in female *Saa3* ^−/−^ mice. (A–B) Triglycerides and (C–D) total cholesterol were measured from fasted plasma after 16 weeks on chow or HFHSC diet. (E–F) Lipoprotein profiles were obtained by fast-phase liquid chromatography (FPLC) of pooled fasted plasma samples taken at sacrifice. n = 6–15 mice per group. *P<0.05 from chow group; #P<0.05 from *Saa3* ^+/+^ controls. WT: *Saa3* ^+/+;^ KO: *Saa3* ^−/−^.

Total triglycerides and cholesterol were measured from fasting plasma from all animals after 16 weeks on diet. As shown in Figure 2A–B, fasting triglycerides were not increased by HFHSC diet, but were significantly reduced in female *Saa3* ^−/−^ mice. Predictably, fasting cholesterol levels increased considerably in all animals fed the HFHSC diet, and were also reduced in female *Saa3* ^−/−^ mice (Figure 2C–D). FPLC from pooled fasting plasma suggests that this decrease in cholesterol can be attributed to female *Saa3* ^+/−^ and *Saa3* ^−/−^ mice having less total LDL (Figure 2F). Notably, there was no improvement in any lipoprotein class in male *Saa3* ^−/−^ mice (Figure 2E).

To determine if the reduced weight gain in the absence of *Saa3* improved glucose metabolism over *Saa3* ^+/+^ controls, glucose tolerance (GT) and insulin tolerance (IT) tests were performed. While the HFHSC diet worsened glucose and insulin tolerance in both males and females (Figure S4 in File S1), there were no differences between *Saa3* ^+/+^ and *Saa3* ^−/−^ mice for either sex. Concurrently, fasting insulin levels were not different between any groups, with no improvements in blood glucose over time.

### Female *Saa3^−/−^* mice demonstrate decreased visceral adipose tissue expression of inflammatory and chemotactic factors

**Figure 3:**
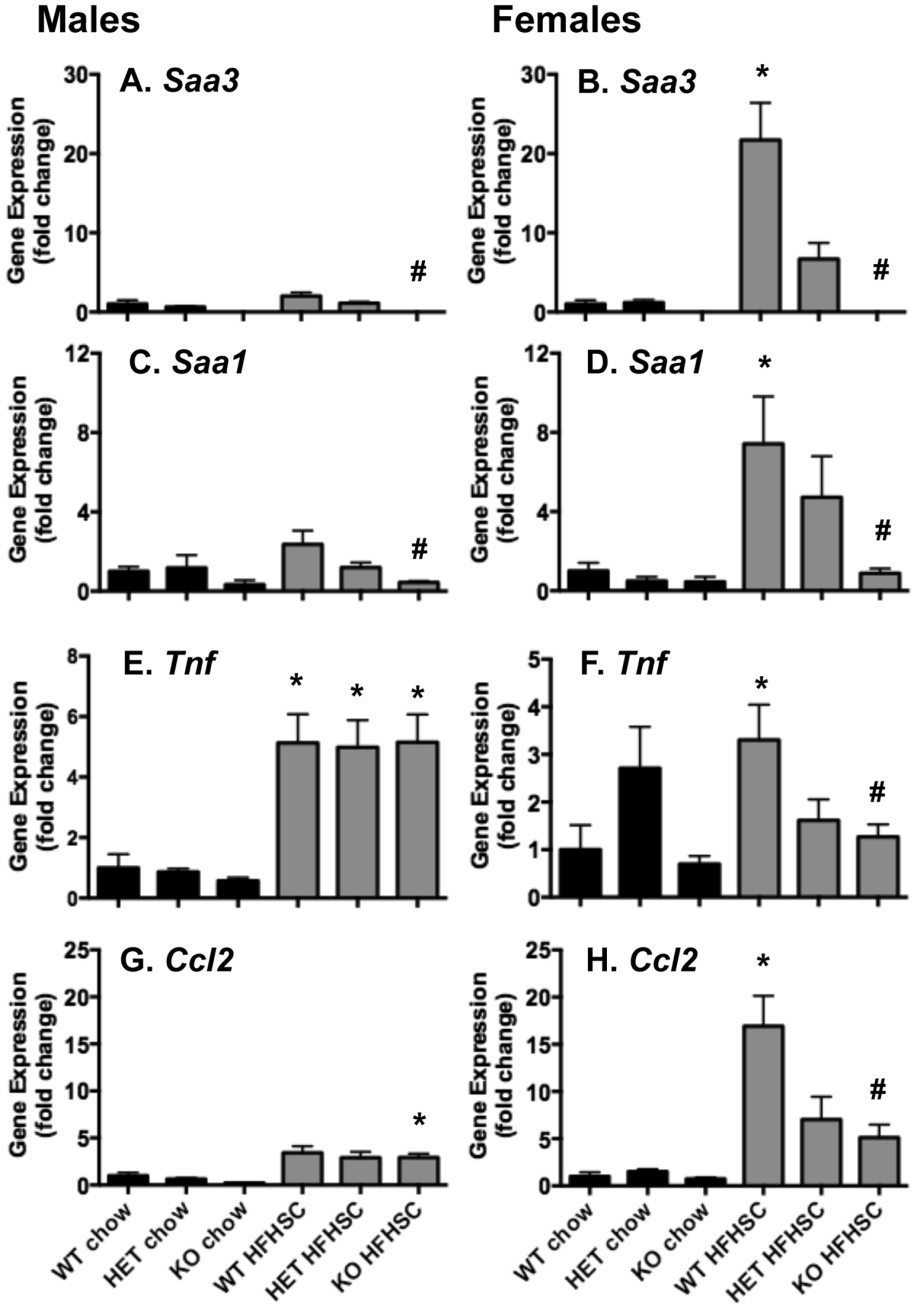
Gonadal white adipose tissue inflammatory and chemotactic gene expression is attenuated in female *Saa3* ^−/−^ mice. (A–H) Total RNA from whole gonadal white adipose tissue (gWAT) was reverse transcribed into cDNA for quantitative PCR analysis. Genes including *Saa3* (A–B), *Saa1* (C–D), *Tnf* (E–F), and *Ccl2* (G–H) are presented, normalized to an internal control gene (*Gapdh*) and presented as fold change from *Saa3* ^+/+^ chow controls. n = 6–15 mice per group. *P<0.05 from chow group; #P<0.05 from *Saa3* ^+/+^ controls. WT: *Saa3* ^+/+;^ HET: *Saa3* ^+/−^; KO: *Saa3* ^−/−^.

Since we have previously shown that *Saa3* expression is increased in visceral and subcutaneous adipose tissue in response to the HFHSC diet in C57BL/6 mice [32], we examined inflammatory and chemotactic gene expression in the same tissues in *Saa3* ^−/−^ mice. As expected, *Saa3* expression was absent in whole gWAT from both male and female *Saa3* ^−/−^ mice, and significantly reduced in female *Saa3* ^+/−^ mice (Figure 3A–B). Although not highly expressed in adipose tissue, *Saa1* was also reduced in *Saa3* ^−/−^ mice (Figure 3C–D). A direct comparison of expression levels of *Saa1* and *Saa3* in gWAT from these mice showed that *Saa3* is ∼300-fold more abundant than *Saa1* in both males and females in *Saa3* ^+/+^ chow-fed mice (Table 1 and Figure S5 in File S1). Furthermore, while both *Saa1* and *Saa3* are induced by the HFHSC diet in gWAT from both sexes, *Saa3* is induced to considerably higher levels than *Saa1*. Furthermore, Table 1 exemplifies the considerably higher induction of *Saa3* by HFHSC diet in female mice than males (15- vs. 2-fold, respectively). Moreover, *Saa3* ^−/−^ mice had no gWAT *Saa3* expression, with only a slight decrease in *Saa1*. This analysis supports the current dogma that visceral fat expresses large amounts of *Saa3*, with little contribution by *Saa1*. Interestingly, while expression levels of *Tnf* and *Ccl2* were unchanged in male gWAT (Figure 3E, G), female *Saa3* ^−/−^ mice showed significant decreases in *Tnf* and *Ccl2* (Figure 3F, H). Notably, *Saa3* ^+/−^ female mice fed HFHSC diet also had reduced expression of *Tnf* and *Ccl2* (Figure 3F, H), suggesting that the absence of only one allele is necessary to reduce inflammatory gene expression. Similar gene expression profiles were seen in whole inguinal white adipose tissue (iWAT) (Figure S6 in File S1), again highlighting a trend towards improved inflammatory and chemotactic gene expression profiles in female *Saa3* ^−/−^ mice, but not in male mice.

**Table 1:** Relative expression of *Saa* subtypes in gWAT.

	gWAT			
	Males		Females	
	*Saa1*	*Saa3*	*Saa1*	*Saa3*
*Saa3* ^+/+^ chow	1	330	1	363
*Saa3* ^−/−^ chow	0	1	0	1
*Saa3* ^+/+^ HFHSC	2	660	3	5460
*Saa3* ^−/−^ HFHSC	0	1	1	3

### Visceral adipose tissue macrophage content is attenuated in female *Saa3* ^−/−^ mice

**Figure 4:**
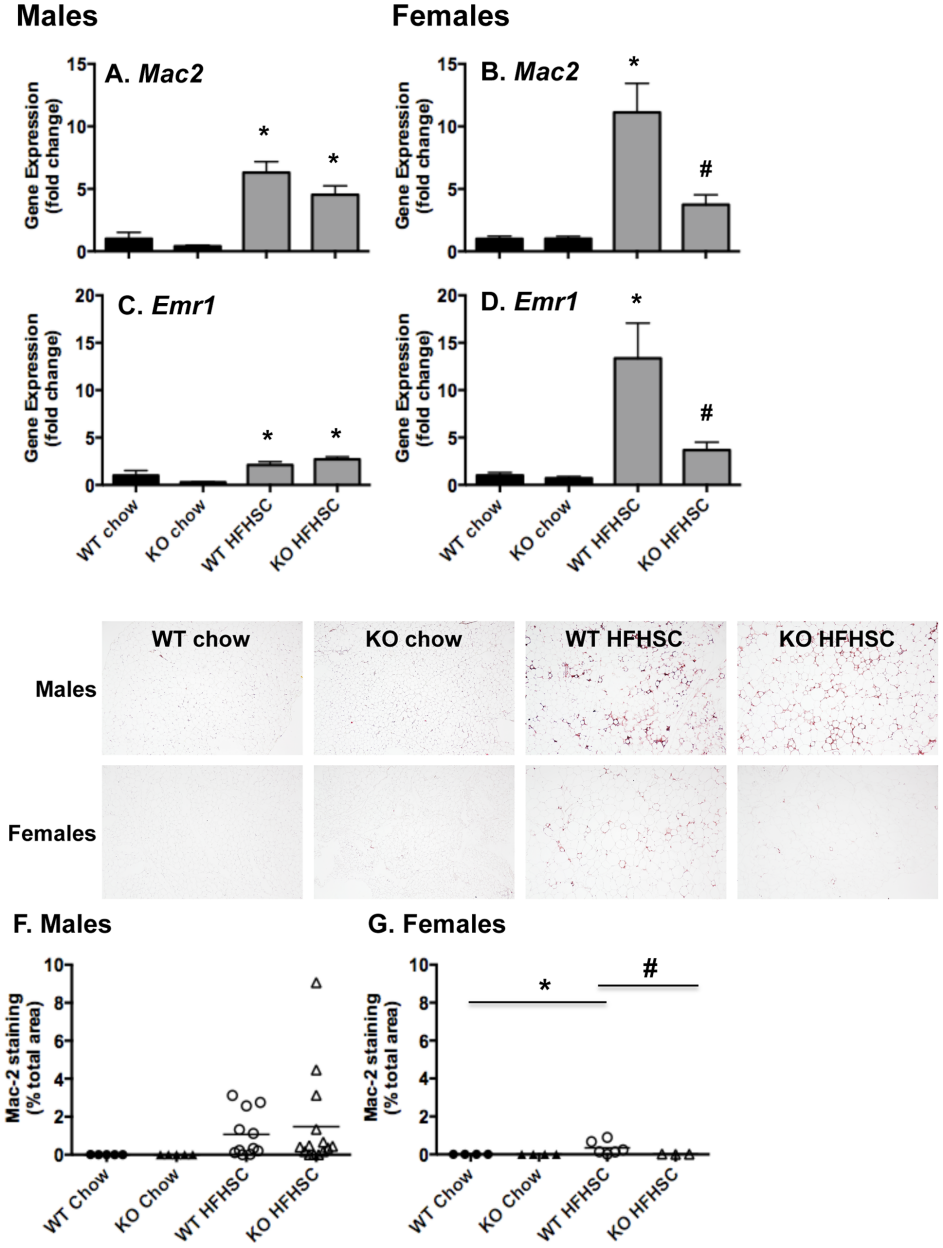
Macrophage content of gonadal white adipose tissue is decreased in female *Saa3* ^−/−^ mice. (A–D) Total RNA from whole gonadal white adipose tissue (gWAT) was reverse transcribed into cDNA for quantitative PCR analysis. Genes including *Mac2* (A–B) and *Emr1* (C–D) are presented, normalized to an internal control gene (*Gapdh*) and presented as fold change from *Saa3* ^+/+^ chow controls. (E–G) gWAT was fixed in formalin and embedded in formalin before sectioning and staining with a Mac2 antibody. (E) Representative images are shown, 10X magnification. (F–G) Quantification of the percentage of total Mac2-stained area in all tissue sections examined. n = 6–15 mice per group. *P<0.05 from chow group; #P<0.05 from *Saa3* ^+/+^ controls. WT: *Saa3* ^+/+;^ KO: *Saa3* ^−/−^.

The macrophage content of white adipose tissue is thought to contribute to the pro-inflammatory state associated with obesity [40]. We and others have shown that SAA3 is chemotactic for macrophages [41]. We therefore examined macrophage accumulation within white adipose tissue in *Saa3* ^−/−^ mice by quantitative real-time PCR for the general macrophage markers *Mac2* and *Emr1* and immunostaining for Mac2. As shown in Figure 4A–D, expression of macrophage markers *Mac2* and *Emr1* is significantly decreased in whole gWAT from female *Saa3* ^−/−^ mice, but not from male *Saa3* ^−/−^ mice when compared to *Saa3* ^+/+^ controls. This is corroborated by decreased Mac2 protein immunostaining in gWAT (Figure 4E–G). Mechanistically, these data are in line with our observation that female *Saa3* ^−/−^ mice have reduced chemotactic factor expression in gWAT (Figure 3H). In subcutaneous iWAT, while *Saa1* and *Saa3* gene expression were reduced in *Saa3* ^−/−^ mice, there were no significant changes in inflammatory cytokine and macrophage gene expression (Figure S6 and S7, respectively, in File S1).

### Liver *Saa* expression and circulating SAA levels are decreased in *Saa3* ^−/−^ mice

**Figure 5:**
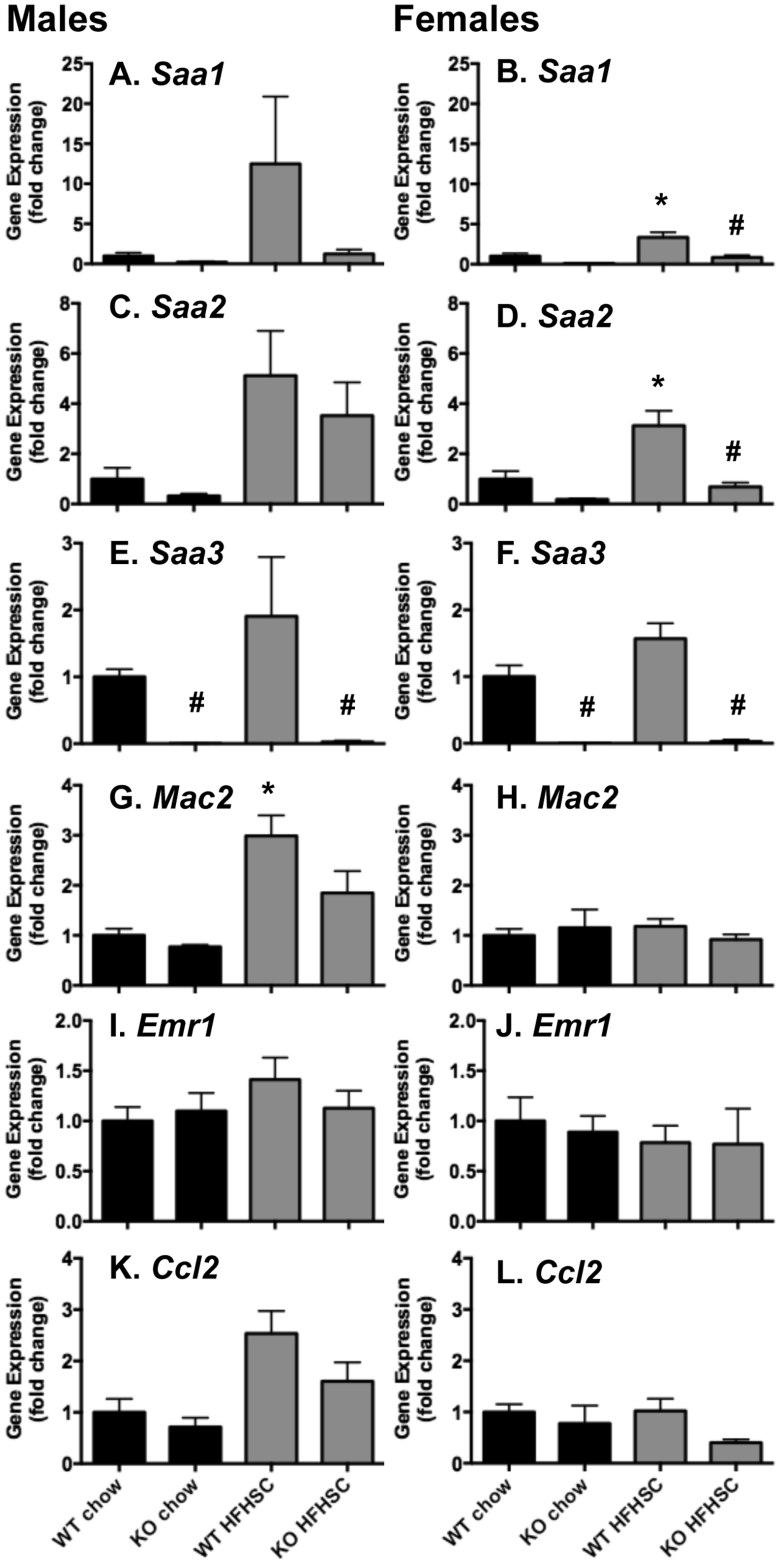
Liver *Saa1* and *Saa2* are attenuated in *Saa3* ^−/−^ mice. Total RNA from whole liver was reverse transcribed into cDNA for quantitative PCR analysis. Genes including *Saa1* (A–B), *Saa2* (C–D), *Saa3* (E–F), *Mac2* (G–H), *Emr1* (I–J), and *Ccl2* (K–L) are presented, normalized to an internal control gene (*Gapdh*) and presented as fold change from *Saa3* ^+/+^ chow controls. n = 6–15 mice per group. *P<0.05 from chow group; #P<0.05 from *Saa3* ^+/+^ controls. WT: *Saa3* ^+/+;^ KO: *Saa3* ^−/−^.

**Figure 6:**
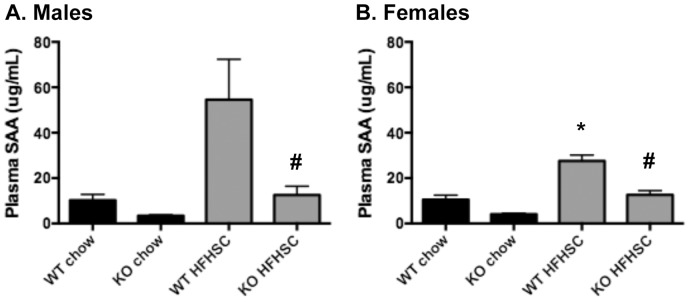
Plasma SAA is decreased in both male and female *Saa3* *^−^* ^/*−*^ mice. (A–B) Total SAA was measured from fasted plasma taken at sacrifice by ELISA. n = 6–15 mice per group. *P<0.05 from chow group; #P<0.05 from *Saa3* ^+/+^ controls. WT: *Saa3* ^+/+;^ KO: *Saa3* ^−/−^.

The murine liver expresses all four SAA subtypes, but is known predominantly to secrete SAA1 and SAA2 into the circulation, primarily associated with HDL particles. Plasma SAA, as an acute phase reactant, is associated with systemic and liver inflammation [17]. To determine the effect of *Saa3* deletion on liver inflammation and lipid metabolism, gene expression analysis was performed. As shown in Figure 5, *Saa1*, *Saa2*, and *Saa3* are all induced in the liver by HFHSC diet, and are all attenuated in *Saa3* ^−/−^ mice of both sexes. This translated to decreased SAA protein in fasting plasma from *Saa3* ^−/−^ mice (Figure 6A–B).

We next determined relative expression levels of the three primary SAA subtypes expressed by the liver, *Saa1–3*, shown in Figure S4C–D in File S1 and Table 2. Contrary to gWAT expression patterns, *Saa* subtypes were expressed in the liver of chow-fed *Saa3* ^+/+^ mice as follows: *Saa2*> *Saa1*> *Saa3*. Moreover, the HFHSC diet further increased expression of *Saa1* and *Saa2* to much higher levels than *Saa3*. Again contrary to expression patterns in gWAT, livers from male mice showed a much higher induction of *Saa1* and *Saa2* than female mice (Table 2). As expected, liver *Saa3* was not expressed by *Saa3* ^−/−^ mice, while *Saa1* and *Saa2* were only slightly decreased in the *Saa3* ^−/−^ males.

**Table 2:** Relative expression of *Saa* subtypes in liver.

	Liver					
	Males			Females		
	*Saa1*	*Saa2*	*Saa3*	*Saa1*	*Saa2*	*Saa3*
*Saa3* ^+/+^ chow	3	16	1	2	39	1
*Saa3* ^−/−^ chow	1	5	0	0	7	0
*Saa3* ^+/+^ HFHSC	42	232	2	9	123	2
*Saa3* ^−/−^ HFHSC	5	57	0	2	27	0

In the liver, there were no changes in expression of macrophage (*Mac2*, *Emr1*) or chemotactic factors (*Ccl2*) between genotypes. In addition, despite improved lipid profiles in female *Saa3* ^−/−^ mice, there were no differences in genes involved in triglyceride synthesis (*Dgat1*), cholesterol synthesis (*Srebp1*), fatty acid synthesis (*Fasn*), or fatty acid oxidation (*Cpt1alpha*) (Figure S8 in File S1). Concurrently, livers from *Saa3^−/−^* mice showed no difference in triglyceride or cholesterol content (Figure S9 in File S1). These results suggest that attenuation of liver SAA by deletion of *Saa3* does not directly impact liver inflammation, lipid synthesis genes, or liver fat content.

## Discussion

In this study, we examined the role of *Saa3* in a mouse model of chronic low-grade inflammation. The absence of *Saa3* had significant effects on adipose tissue, liver, and systemic inflammation with diet-induced obesity. In addition, we have shown that deletion of *Saa3* leads to diminished weight gain in mice on a HFHSC diet that cannot be attributed to changes in fat and lean body mass ratios or basal metabolism. Moreover, the blunted weight gain in *Saa3* ^−/−^ mice did not improve the insulin resistant phenotype generated by the HFHSC diet. However, the absence of *Saa3* did improve systemic lipid and lipoprotein profiles, as well as adipose tissue-specific inflammation and macrophage content in female mice. Finally, the loss of *Saa3* also attenuated liver-specific *Saa1* and *Saa2* expression and SAA secretion into the blood, suggesting a novel mechanism by which adipose tissue SAA could impact hepatic SAA production.

Human obesity falls into two major classes- “metabolically more healthy” and “metabolically less healthy” [42]. The latter associates more strongly with visceral adiposity, inflammation, dyslipidemia, hypertension, and dysglycemia (i.e., features of the metabolic syndrome [43]), as well as an increased risk of cardiovascular disease [44]. We have found that mice fed a HFHSC diet have many features of the metabolic syndrome in humans [32]. These include local (adipose tissue) and systemic inflammation (as assessed by an increase in the circulating inflammatory marker SAA), dyslipidemia, insulin resistance and mild elevations in fasting glucose levels [32]. Our findings in mice suggest that strategies for reducing adipose tissue inflammation and macrophage accumulation in adipose tissue could have downstream benefits on lipids and systemic inflammation.

The observation that both male and female *Saa3* ^−/−^ mice had blunted weight gain on the HFHSC diet, but only females showed improved adipose tissue inflammation and circulating lipid profile, suggests that these changes in adipose tissue and circulating lipids could be due to enhanced weight loss in females, the specific loss of *Saa3,* or the female gender itself. As a future direction, weight-matched control animals will help determine if the extra weight lost by females contributed to this phenotype. Wild type female mice showed a more robust HFHSC diet-induced increase in *Saa3* expression in gWAT than males (Table 1), possibly due to lower baseline levels of adipose tissue *Saa1* and *Saa3* expression. Moreover, female mice of all genotypes gained less weight on HFHSC diet than male mice, which could have an impact on local inflammation and systemic lipids. A future direction to address this possibility would be to compare weight-matched male and female *Saa3* ^+/+^ and *Saa3* ^−/−^ mice fed the HFHSC diet. The reason for the reduced weight gain in both male and female *Saa3^−/−^* mice is not clear since there were no detectable differences in resting metabolic rate. It is conceivable that our methods were not sufficiently sensitive to pick up small differences in energy expenditure or food intake, which could be studied in future, detailed metabolic cage studies.

A major finding in this study was that SAA3 appears to drive adipose tissue inflammation and macrophage accumulation in females, but not males. This sexual dimorphism could be at least partially explained by the significant decrease in *Ccl2* expression in gWAT of female *Saa3* ^−/−^ mice, while *Ccl2* levels remain elevated in male *Saa3* ^−/−^ mice. This provides further evidence that both *Saa3* and *Ccl2* are required for monocyte recruitment into adipose tissue, and when one is absent the other can compensate for any loss in chemotaxis. This has been corroborated by Weisberg et al., who showed that mice lacking *Ccr2*, the receptor for *Ccl2*, also have reduced but not ablated adipose tissue macrophage content [33]. Our previous studies in cultured adipocytes also support this notion, as silencing both *Saa3* and *Ccl2* had a synergistic effect on decreasing monocyte chemotaxis [29].

It has previously been shown that SAA indirectly impacts MCP-1 expression in HUVECs [45], HepG2 and H22 cells [46], and human monocytes [47] through p38-dependent mechanisms. p38 has been implicated in promoting inflammatory responses in disease states such as rheumatoid arthritis, inflammatory bowel disease, and diabetes [48]–[50]. Recently, p38 was shown to have sexually dimorphic functions related to multiple sclerosis [51], wherein pharmacologic inhibition of p38 ameliorated symptoms of multiple sclerosis in female mice, but not male mice. Taken together, it could be possible that the sexual dimorphic nature of p38 could influence SAA-induced *Ccl2* expression in female mice but not males in our study. In addition, there is some evidence that estrogen confers some protection against high fat diet-induced systemic and adipose tissue inflammation [52], [53], which could contribute to reduced *Ccl2* expression [54], [55]. Lower baseline *Ccl2* expression in the gWAT of female chow-fed *Saa3* ^+/+^ mice compared to their male counterparts supports this notion. It would now be of great interest to determine if the phenotype observed in female *Saa3* ^−/−^ mice could be enhanced by generating a mouse lacking both *Saa3* and *Ccl2*.

Recent work by Sjöholm et al. also supports a sexual dimorphic role of SAA [56]. The authors of this study examined body composition, systemic inflammation, insulin resistance, and adipocyte size in obese diabetic and non-diabetic cohorts of men and women. They showed that plasma SAA correlated more strongly with systemic inflammatory markers such as CRP and IL-6 in women than in men. Moreover, plasma SAA correlated positively with subcutaneous adipocyte size in women only. The authors speculated that there seems to be an unknown sexual dimorphic role for SAA in the context of obesity, which is also supported by our study.

It was previously believed that obesity-induced adipose tissue macrophage accumulation, specifically the polarization of macrophages towards an inflammatory phenotype, contributed to insulin resistance [57]. However, recent evidence suggests that immune cell populations such as macrophages have very little impact on glucose homeostasis in obese conditions. Montes et al. recently showed that attenuating T cell-mediated macrophage accumulation in obese adipose tissue showed no improvement in insulin resistance [58]. In addition, recent work in which transgenic male mice have been engineered to express human SAA1 in intra-abdominal adipose tissue showed no negative effects on insulin resistance [59]. In the current study, we support this theme by showing that the absence of adipose tissue SAA in female *Saa3* ^−/−^defficient mice does not affect glucose metabolism, despite decreasing visceral adipose tissue macrophage content.

A major finding in this study was the effect of *Saa3* deletion on plasma triglycerides, cholesterol, and lipoprotein profiles in female mice. Total triglyceride and cholesterol levels were comparable between male and female mice, but only female *Saa3* ^−/−^ mice showed improvements in these lipids. In addition, males appeared to have more LDL and less HDL than females (Figure 2E–F). Work recently published by Ahlin et al. in which recombinant human SAA1 was overexpressed in adipose tissue from ApoE^−/−^ mice showed that despite increases in plasma SAA, there were no changes in lipoprotein profiles, but only male mice were used in that study [60]. Moreover, a recent study by de Beer et al. showed that deletion of SAA1/2 from ApoE^−/−^ mice does not improve lipoprotein profiles in either male or female mice [61]. Taken together, these two studies suggest that SAA1, whether expressed from adipose tissue or liver, has little effect on lipoprotein metabolism. Therefore, the improvements in female lipoproteins seen in the absence of *Saa3* in our study suggests that liver SAA3 could play an unknown but important role in lipoprotein metabolism. Interestingly, male *Saa3* ^−/−^ mice showed reduced liver size. Because there were no measureable differences in liver cholesterol or triglycerides and no apparent differences in liver histology, we speculate that the reduced liver size in male *Saa3^−/−^* mice is consistent with the reduced body weight of these mice.

Another potentially significant finding in this study is that deletion of *Saa3^−/−^* from both male and female mice led to decreased adipose tissue and liver *Saa* expression as well as circulating SAA levels. This was a surprising finding, given that only female *Saa3* ^−/−^ mice had reduced adipose tissue inflammation and macrophage content. This would suggest that chronically elevated SAA3 could directly or indirectly impact expression and secretion of SAA. It is also conceivable that the reduced SAA levels, mainly SAA1 and SAA2, could have *resulted* in the phenotype seen (reduced weight gain in both males and females and reduced adipose tissue inflammation and lipoprotein changes in females), although it is widely believed that weight loss results in reduced circulating SAA levels and reduced adipose tissue inflammation, rather than the other way around [62]–[64].

In conclusion, we have shown that the absence of *Saa3* confers some protection against HFHSC-induced weight gain, systemic inflammation, hyperlipidemia, and white adipose tissue inflammation in female mice. We speculate that the sexual dimorphic effect seen with deletion of *Saa3* could hold clues regarding its effect on adipose tissue inflammation and macrophage content, and that both SAA3 and MCP-1 are required for efficient inflammatory cell chemotaxis. Future directions made possible by this study would be to examine global *Saa3* deletion in the context of atherosclerosis, given the improved lipid phenotype in females. This could be achieved by generating double *Saa3^−/−^*/*Ldlr^−/−^* mice. Furthermore, it is now prudent to tease apart the individual contributions of *Saa3* from adipocytes and macrophages by generating cell-specific knock out mice.

## Supporting Information

File S1 **Supplementary figures S1–S9.** Contains figures showing additional data that was not included in the main text. **Figure S1. Deletion of** ***Saa3*** **does not alter body composition.** Male and female *Saa3* ^+/+^ (WT) and *Saa3* ^−/−^ (KO) mice were fed either chow or a high fat high sucrose diet with added cholesterol (HFHSC) for 16 weeks. (A–B) Total fat and (C–D) lean mass were estimated using quantitative magnetic resonance spectroscopy after 11 weeks on diet, and expressed as a percentage of total body mass. n = 6–15 mice per group. *P<0.05 from chow group. **Figure S2. Deletion of** ***Saa3*** **does not alter basal metabolism or food intake in females.** Male and female *Saa3* ^+/+^ (WT) and *Saa3* ^−/−^ (KO) mice were fed either chow or a high fat high sucrose diet with added cholesterol (HFHSC) for 16 weeks. (A) V_2_, (B) Vc_2_, (C) respiratory quotient (RQ), (D) heat production, (E) food intake, and (F) activity were calculated using an indirect calorimeter after 11 weeks on diet. n = 6–15 mice per group. *P<0.05 from chow group. **Figure S3. Gonadal adipocyte size is not altered by deletion of** ***Saa3*** **.** Male and female *Saa3* ^+/+^ (WT) and *Saa3* ^−/−^ (KO) mice were fed either chow or a high fat high sucrose diet with added cholesterol (HFHSC) for 16 weeks. Sections of gonadal adipose tissue were stained with Movat's Pentacrhome, and adipocyte size was estimated using Image Pro Plus/Media Cybernectics software. n = 6–15 mice per group. *P<0.05 from chow group. **Figure S4. Glucose homeostasis is not improved by deletion of** ***Saa3*** **.** Male and female *Saa3* ^+/+^ (WT) and *Saa3* ^−/−^ (KO) mice were fed either chow or a high fat high sucrose diet with added cholesterol (HFHSC) for 16 weeks. (A–B) Fasting blood glucose was measured every 4 weeks on diet. (C–F) Glucose tolerance tests (GTT) were performed after 14 weeks on diet. Insulin was measured at the 30-minute time point. (G–H) Insulin tolerance tests (ITT) were performed after 15 weeks on diet. n = 6–15 mice per group. *P<0.05 from chow group, #P<0.05 from *Saa3* ^+/+^ controls. **Figure S5. Relative expression of Saa subtypes in epididymal white adipose tissue (eWAT) and liver.** Male and female *Saa3* ^+/+^ (WT) and *Saa3* ^−/−^ (KO) mice were fed either chow or a high fat high sucrose diet with added cholesterol (HFHSC) for 16 weeks. (A–B)eWAT and (C–D) liver were harvested at sacrifice, and *Saa1* and *Saa3* expression was quantified by RT-PCR. Results are presented normalized to *Saa1* expression in WT mice for eWAT subtype comparison, and normalized to *Saa3* expression in WT mice for liver subtype comparison, +/− SEM. n = 6–15 mice per group. *P<0.05 from chow group, #P<0.05 from *Saa3* ^+/+^ controls. **Figure S6. Inflammatory gene expression profiles in inguinal white adipose tissue (iWAT).** Male and female *Saa3* ^+/+^ (WT) and *Saa3* ^−/−^ (KO) mice were fed either chow or a high fat high sucrose diet with added cholesterol (HFHSC) for 16 weeks. iWAT was harvested at sacrifice and (A–B) *Saa3*, (C–D) *Saa1*, (E–F) *Tnf*, and (G–H) *Ccl2* expression was quantified by RT-PCR. Results are presented normalized to WT chow mice, +/− SEM. n = 6–15 mice per group. *P<0.05 from chow group, #P<0.05 from *Saa3* ^+/+^ controls. **Figure S7. Inflammatory gene expression profiles in inguinal white adipose tissue (iWAT).** Male and female *Saa3* ^+/+^ (WT) and *Saa3* ^−/−^ (KO) mice were fed either chow or a high fat high sucrose diet with added cholesterol (HFHSC) for 16 weeks. iWAT was harvested at sacrifice and (A–B) *Mac2* and (C–D) *Emr1* expression was quantified by RT-PCR. Results are presented normalized to WT chow mice, +/− SEM. n = 6–15 mice per group. *P<0.05 from chow group. **Figure S8. Lipid synthesis genes are not altered by deletion of** ***Saa3*** **in liver.** Male and female *Saa3* ^+/+^ (WT) and *Saa3* ^−/−^ (KO) mice were fed either chow or a high fat high sucrose diet with added cholesterol (HFHSC) for 16 weeks. Liver was harvested at sacrifice and (A–B) *Dgat,* (C–D) *Srebp1,* (E–F) Fasn, and (G–H) *Cpt1α* expression was quantified by RT-PCR. Results are presented normalized to WT chow mice, +/− SEM. n = 6–15 mice per group. **Figure S9. Liver lipids and histology**. (A–D) Lipids were extracted from previously frozen liver samples using the Folch method. Cholesterol (Chol.: A, C) and triglycerides (TG: B, D) were quantified. E. Representative images of Masson's trichrome-stained liver sections from male and female, WT (*Saa3^+/+^*) and KO (*Saa3* ^−/−^) mice after 16 weeks of chow or HFHSC diet. Scale bar  = 200 µm.
(PDF)
