# Supplementary material for: Deletion of Serum Amyloid A3 Improves High Fat High Sucrose Diet-Induced Adipose Tissue Inflammation and Hyperlipidemia in Female Mice
Source: PLoS One. 2014 Sep 24;9(9):e108564. doi: 10.1371/journal.pone.0108564 (PMC4177399; doi:10.1371/journal.pone.0108564)
Supplement: File S1 — Supplementary figures S1–S9. Contains figures showing additional data that was not included in the main text. Figure S1. Deletion of Saa3 does not alter body composition. Male and female Saa3 +/+ (WT) and Saa3 −/− (KO) mice were fed either chow or a high fat high sucrose diet with added cholesterol (HFHSC) for 16 weeks. (A–B) Total fat and (C–D) lean mass were estimated using quantitative magnetic resonance spectroscopy after 11 weeks on diet, and expressed as a percentage of total body mass. n = 6–15 mice per group. *P<0.05 from chow group. Figure S2. Deletion of Saa3 does not alter basal metabolism or food intake in females. Male and female Saa3 +/+ (WT) and Saa3 −/− (KO) mice were fed either chow or a high fat high sucrose diet with added cholesterol (HFHSC) for 16 weeks. (A) V2, (B) Vc2, (C) respiratory quotient (RQ), (D) heat production, (E) food intake, and (F) activity were calculated using an indirect calorimeter after 11 weeks on diet. n = 6–15 mice per group. *P<0.05 from chow group. Figure S3. Gonadal adipocyte size is not altered by deletion of Saa3 . Male and female Saa3 +/+ (WT) and Saa3 −/− (KO) mice were fed either chow or a high fat high sucrose diet with added cholesterol (HFHSC) for 16 weeks. Sections of gonadal adipose tissue were stained with Movat's Pentacrhome, and adipocyte size was estimated using Image Pro Plus/Media Cybernectics software. n = 6–15 mice per group. *P<0.05 from chow group. Figure S4. Glucose homeostasis is not improved by deletion of Saa3 . Male and female Saa3 +/+ (WT) and Saa3 −/− (KO) mice were fed either chow or a high fat high sucrose diet with added cholesterol (HFHSC) for 16 weeks. (A–B) Fasting blood glucose was measured every 4 weeks on diet. (C–F) Glucose tolerance tests (GTT) were performed after 14 weeks on diet. Insulin was measured at the 30-minute time point. (G–H) Insulin tolerance tests (ITT) were performed after 15 weeks on diet. n = 6–15 mice per group. *P<0.05 from chow group, #P<0.05 from Saa3 +/+ contro [file pone.0108564.s001.pdf]

Figure S1

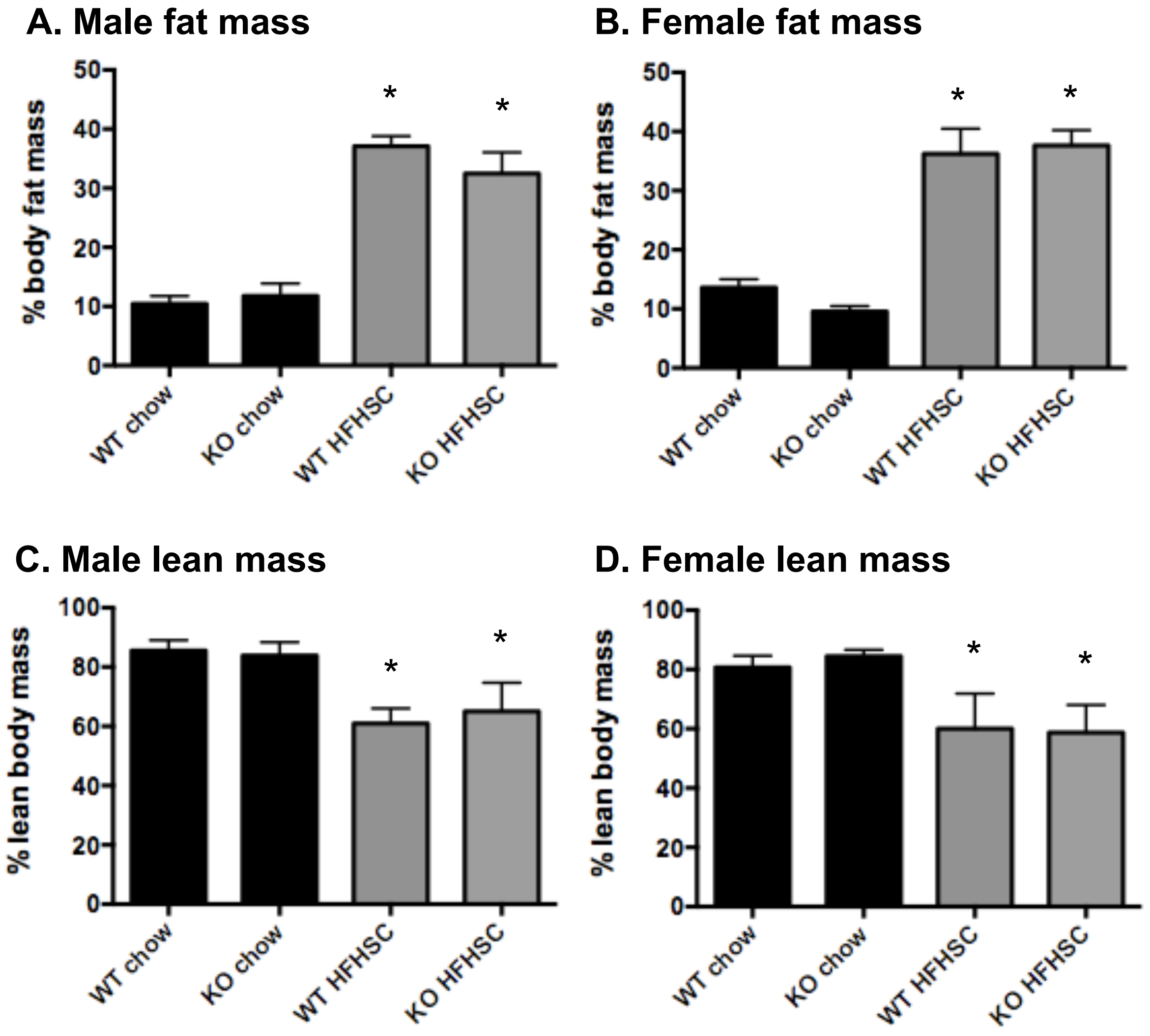

Figure S1. Deletion of *Saa3* does not alter body composition. Male and female *Saa3*$^{+/+}$ (WT) and *Saa3*$^{-/-}$ (KO) mice were fed either chow or a high fat high sucrose diet with added cholesterol (HFHSC) for 16 weeks. (A-B) Total fat and (C-D) lean mass were estimated using quantitative magnetic resonance spectroscopy after 11 weeks on diet, and expressed as a percentage of total body mass. n=6-15 mice per group. *P<0.05 from chow group.

Figure S2

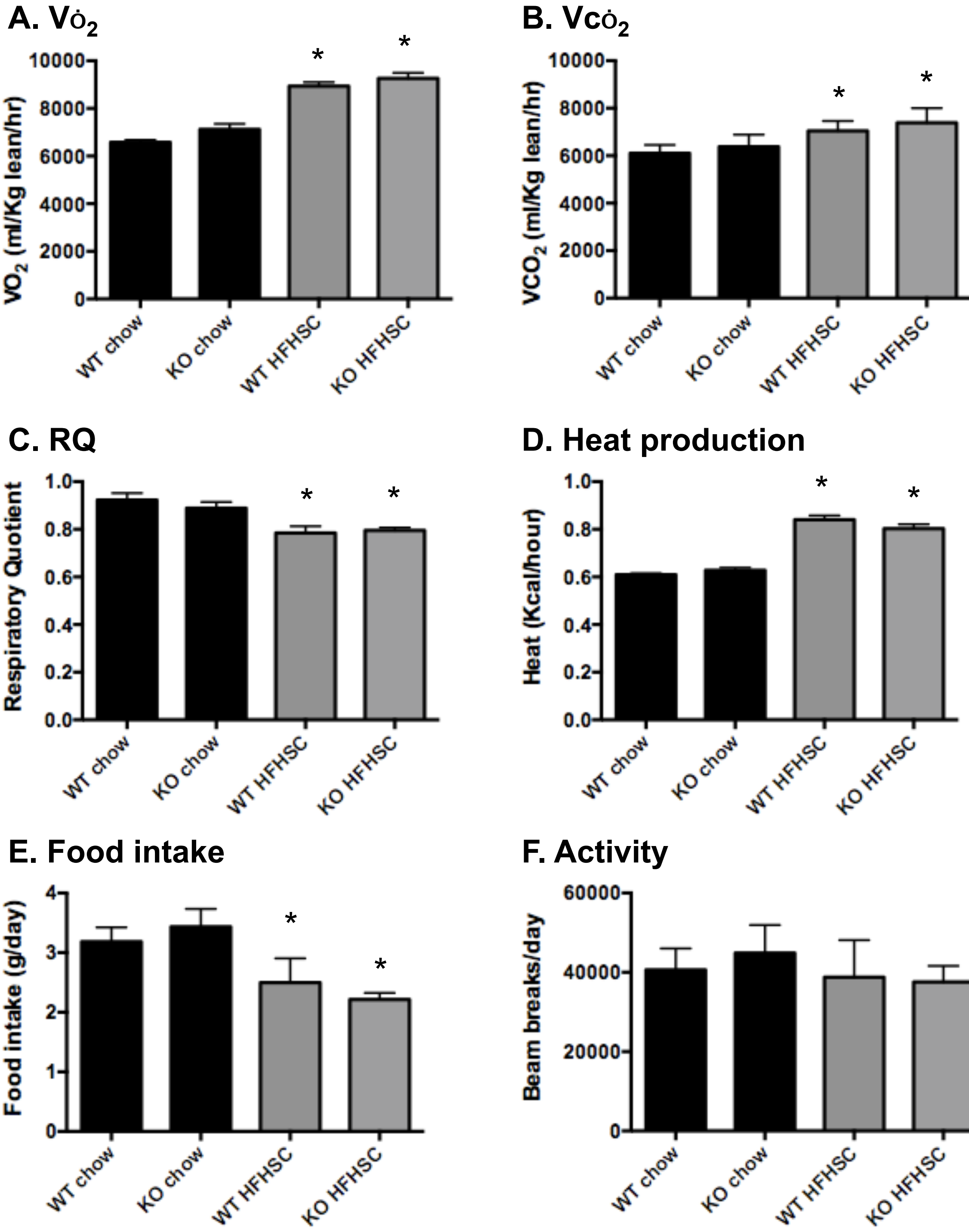

Figure S2. Deletion of *Saa3* does not alter basal metabolism or food intake in females. Male and female *Saa3*$^{+/+}$ (WT) and *Saa3*$^{-/-}$ (KO) mice were fed either chow or a high fat high sucrose diet with added cholesterol (HFHSC) for 16 weeks. (A) $V\dot{o}_2$, (B) $Vc\dot{o}_2$, (C) respiratory quotient (RQ), (D) heat production, (E) food intake, and (F) activity were calculated using an indirect calorimeter after 11 weeks on diet. n=6-15 mice per group. *P<0.05 from chow group.

Figure S3

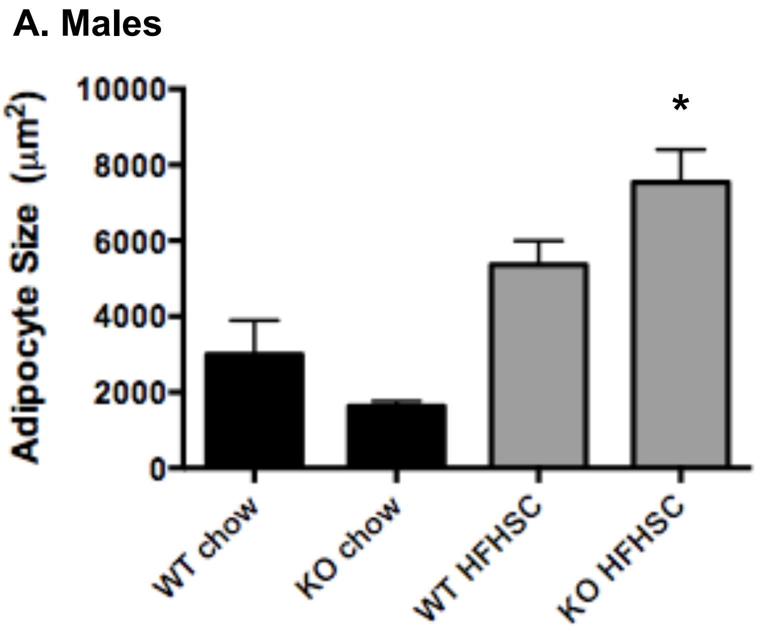

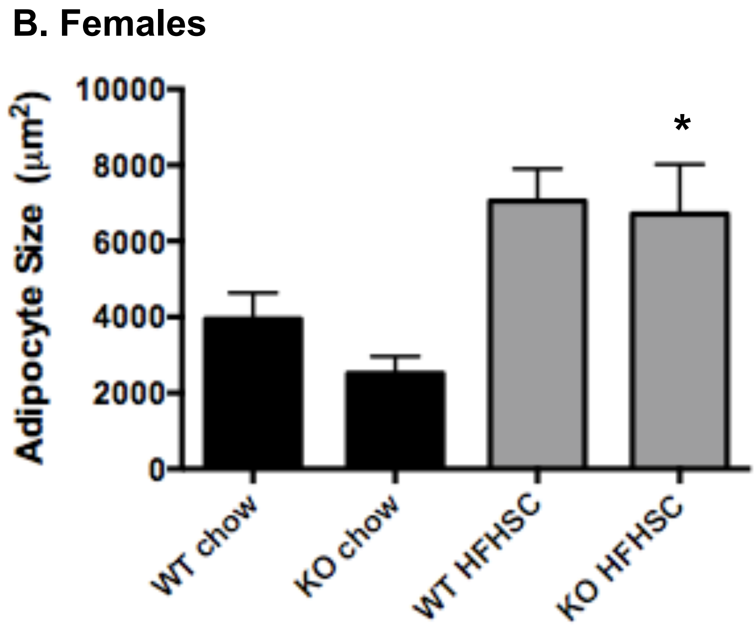

Figure S3. Gonadal adipocyte size is not altered by deletion of *Saa3*. Male and female *Saa3*$^{+/+}$ (WT) and *Saa3*$^{-/-}$ (KO) mice were fed either chow or a high fat high sucrose diet with added cholesterol (HFHSC) for 16 weeks. Sections of gonadal adipose tissue were stained with Movat's Pentacrhome, and adipocyte size was estimated using Image Pro Plus/Media Cybernectics software. n=6-15 mice per group. *P<0.05 from chow group.

Figure S4

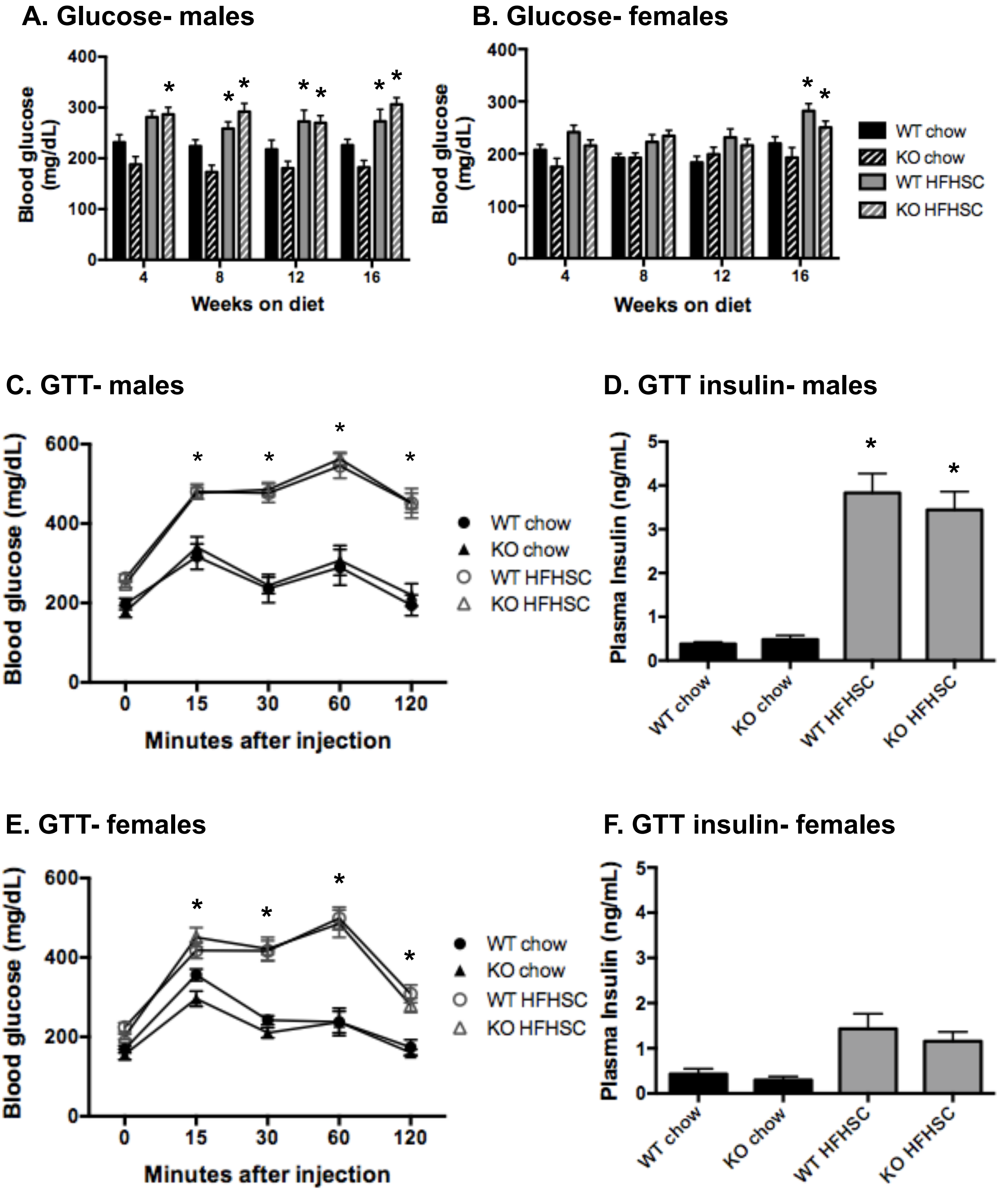
A. Glucose- males
B. Glucose- females
Blood glucose (mg/dL)
Weeks on diet
WT chow
KO chow
WT HFHSC
KO HFHSC
C. GTT- males
D. GTT insulin- males
E. GTT- females
F. GTT insulin- females
Blood glucose (mg/dL)
Minutes after injection
Plasma Insulin (ng/mL)

Figure S4 cont.

G. ITT- males

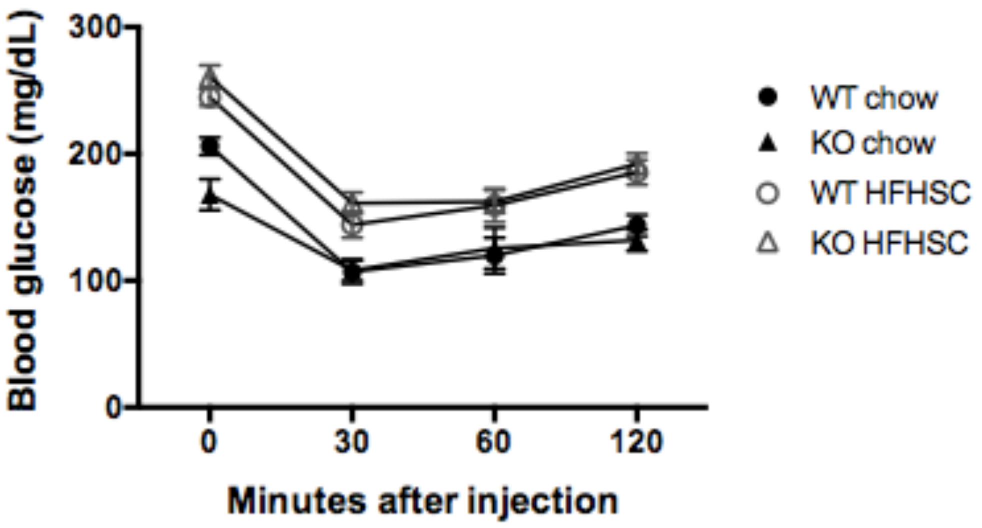

H. ITT- females

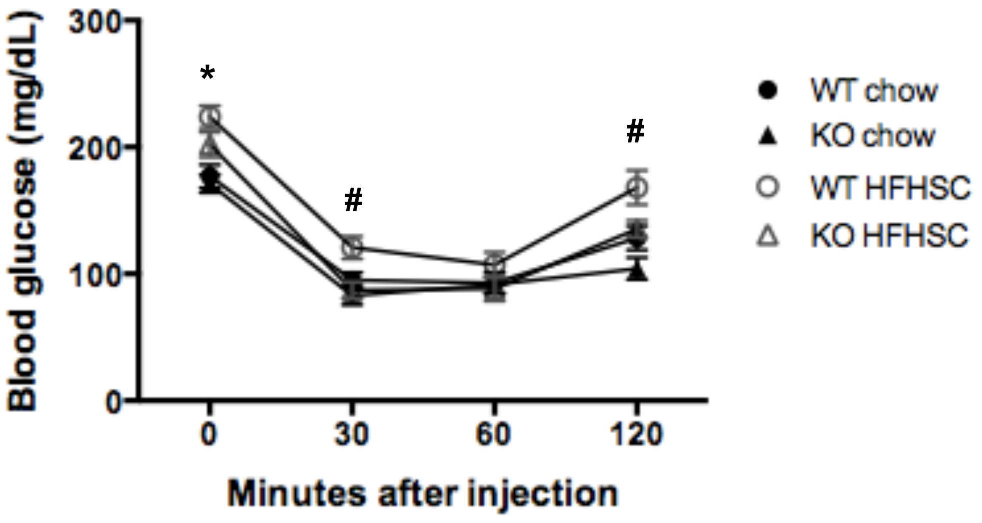

Figure S4. Glucose homeostasis is not improved by deletion of *Saa3*. Male and female *Saa3*$^{+/+}$ (WT) and *Saa3*$^{-/-}$ (KO) mice were fed either chow or a high fat high sucrose diet with added cholesterol (HFHSC) for 16 weeks. (A-B) Fasting blood glucose was measured every 4 weeks on diet. (C-F) Glucose tolerance tests (GTT) were performed after 14 weeks on diet. Insulin was measured at the 30-minute time point. (G-H) Insulin tolerance tests (ITT) were performed after 15 weeks on diet. n=6-15 mice per group. *P<0.05 from chow group, #P<0.05 from *Saa3*$^{+/+}$ controls.

Figure S5

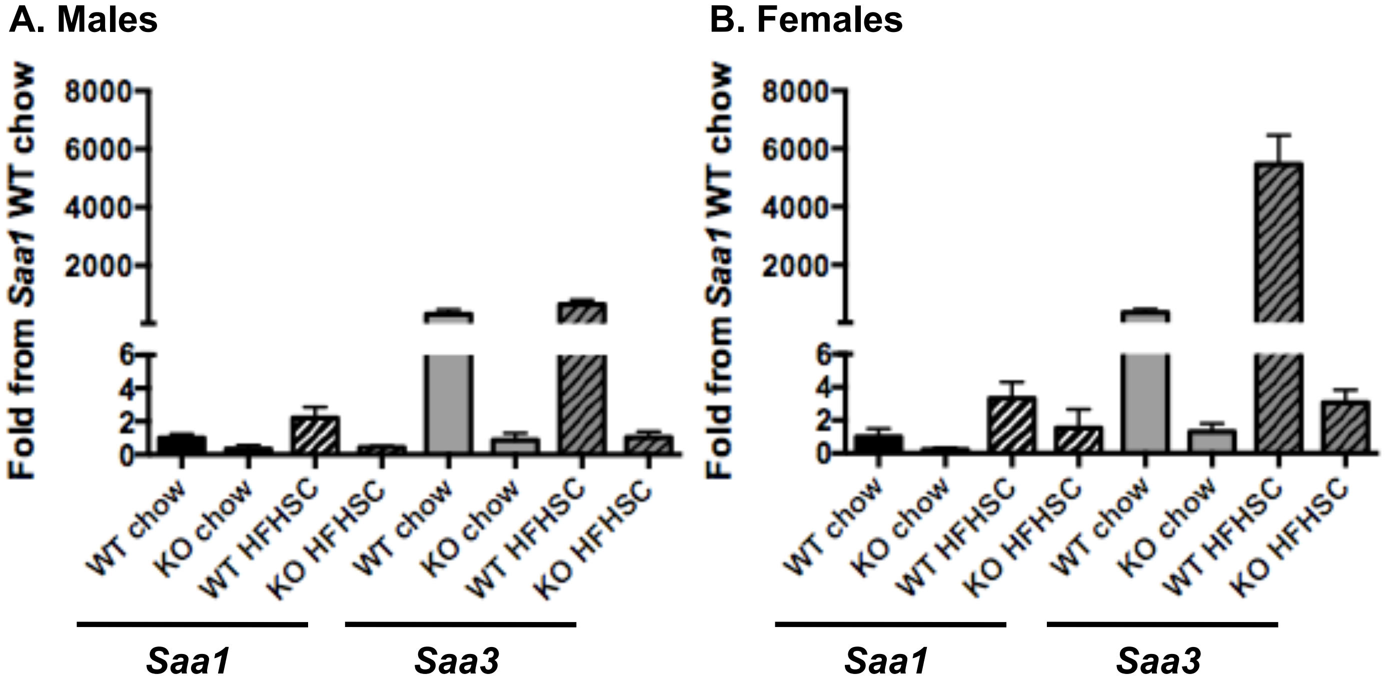

Figure S5. Relative expression of Saa subtypes in epididymal white adipose tissue (eWAT) and liver. Male and female *Saa3*$^{+/+}$ (WT) and *Saa3*$^{-/-}$ (KO) mice were fed either chow or a high fat high sucrose diet with added cholesterol (HFHSC) for 16 weeks. (A-B) eWAT and (C-D) liver were harvested at sacrifice, and *Saa1* and *Saa3* expression was quantified by RT-PCR. Results are presented normalized to *Saa1* expression in WT mice for eWAT subtype comparison, and normalized to *Saa3* expression in WT mice for liver subtype comparison, +/- SEM. n=6-15 mice per group. *P<0.05 from chow group, #P<0.05 from *Saa3*$^{+/+}$ controls.

Figure S5 cont.

C. Males- Liver

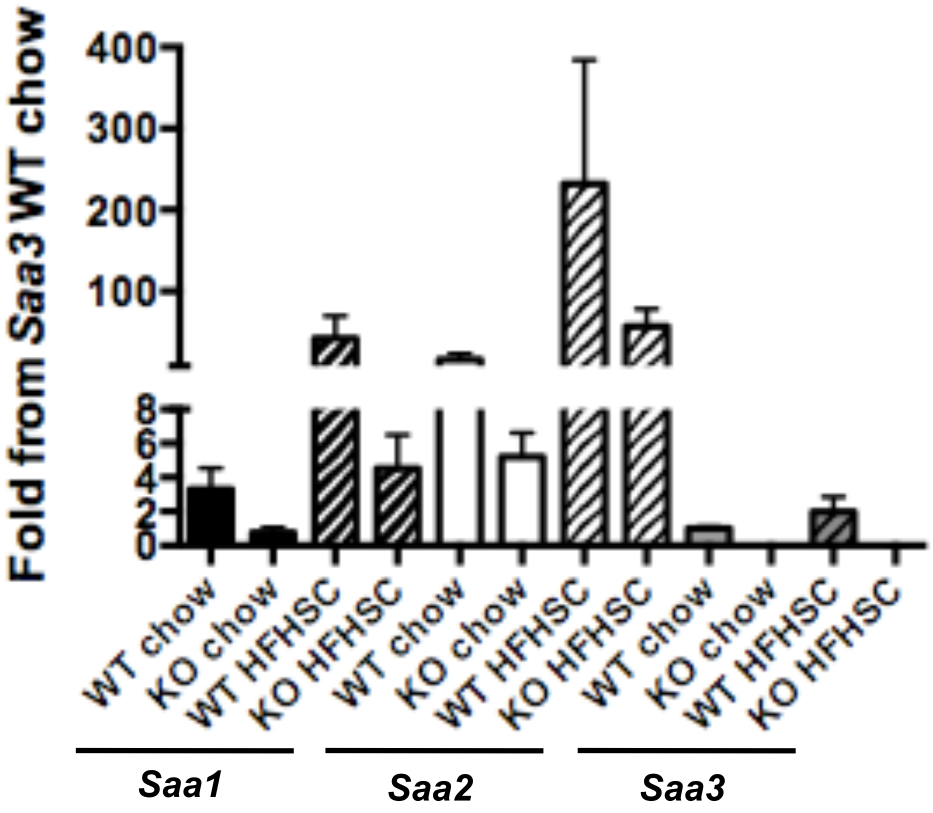

D. Females- Liver

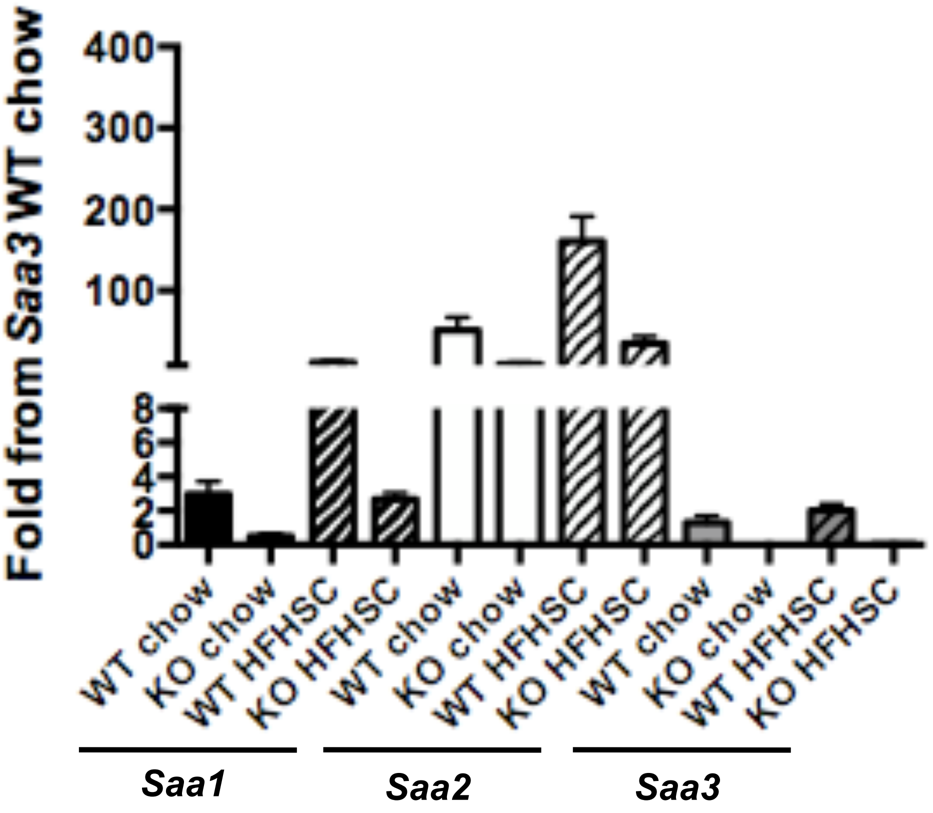

Figure S6

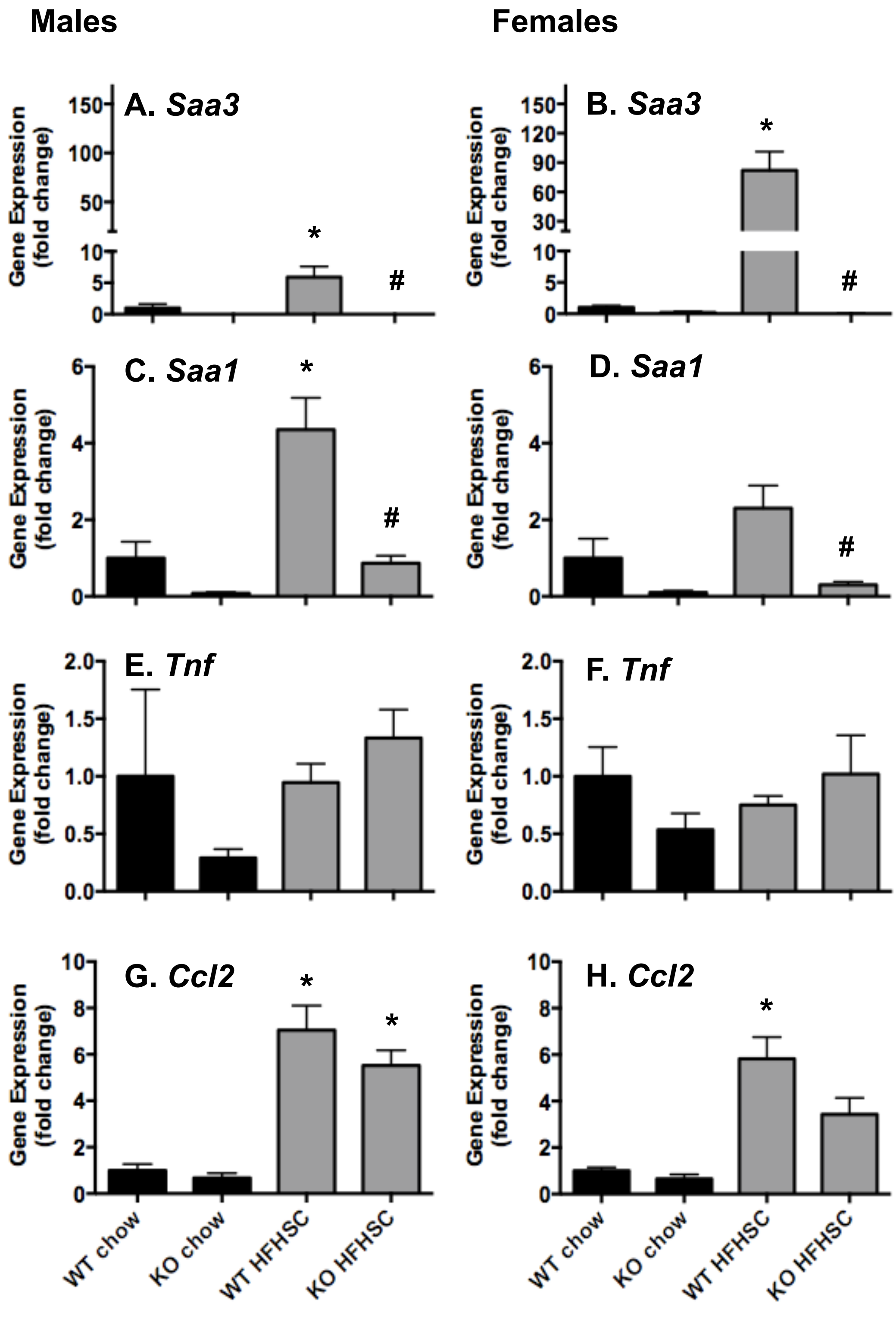

Figure S6. Inflammatory gene expression profiles in inguinal white adipose tissue (iWAT). Male and female *Saa3*$^{+/+}$ (WT) and *Saa3*$^{-/-}$ (KO) mice were fed either chow or a high fat high sucrose diet with added cholesterol (HFHSC) for 16 weeks. iWAT was harvested at sacrifice and (A-B) *Saa3*, (C-D) *Saa1*, (E-F) *Tnf*, and (G-H) *Ccl2* expression was quantified by RT-PCR. Results are presented normalized to WT chow mice, +/- SEM. n=6-15 mice per group. *P<0.05 from chow group, #P<0.05 from *Saa3*$^{+/+}$ controls.

Figure S7

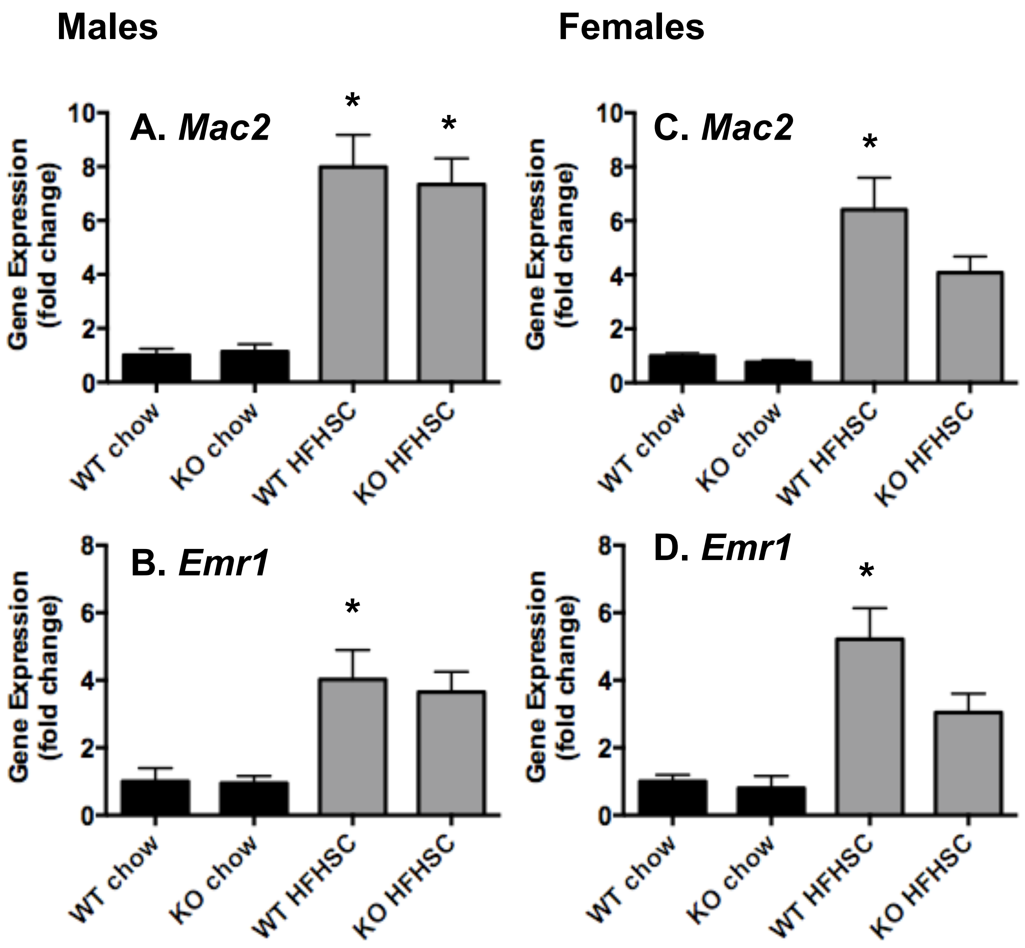

Figure S7. Inflammatory gene expression profiles in inguinal white adipose tissue (iWAT). Male and female *Saa3*$^{+/+}$ (WT) and *Saa3*$^{-/-}$ (KO) mice were fed either chow or a high fat high sucrose diet with added cholesterol (HFHSC) for 16 weeks. iWAT was harvested at sacrifice and (A-B) *Mac2* and (C-D) *Emr1* expression was quantified by RT-PCR. Results are presented normalized to WT chow mice, +/- SEM. n=6-15 mice per group. *P<0.05 from chow group.

Figure S8

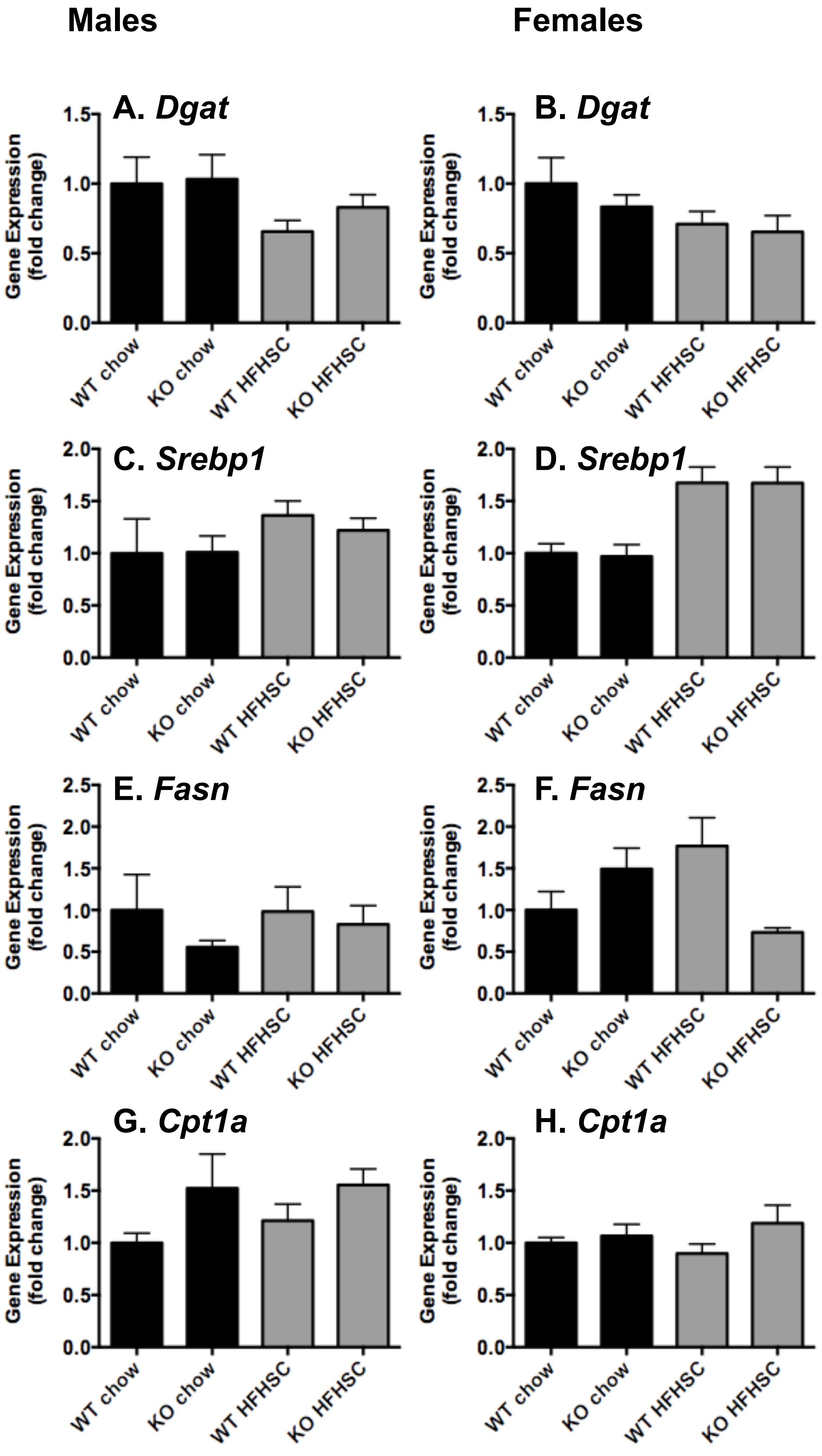

Figure S8. Lipid synthesis genes are not altered by deletion of *Saa3* in liver. Male and female *Saa3*$^{+/+}$ (WT) and *Saa3*$^{-/-}$ (KO) mice were fed either chow or a high fat high sucrose diet with added cholesterol (HFHSC) for 16 weeks. Liver was harvested at sacrifice and (A-B) *Dgat,* (C-D) *Srebp1,* (E-F) Fasn, and (G-H) *Cpt1α* expression was quantified by RT-PCR. Results are presented normalized to WT chow mice, +/- SEM. n=6-15 mice per group.

Figure S9

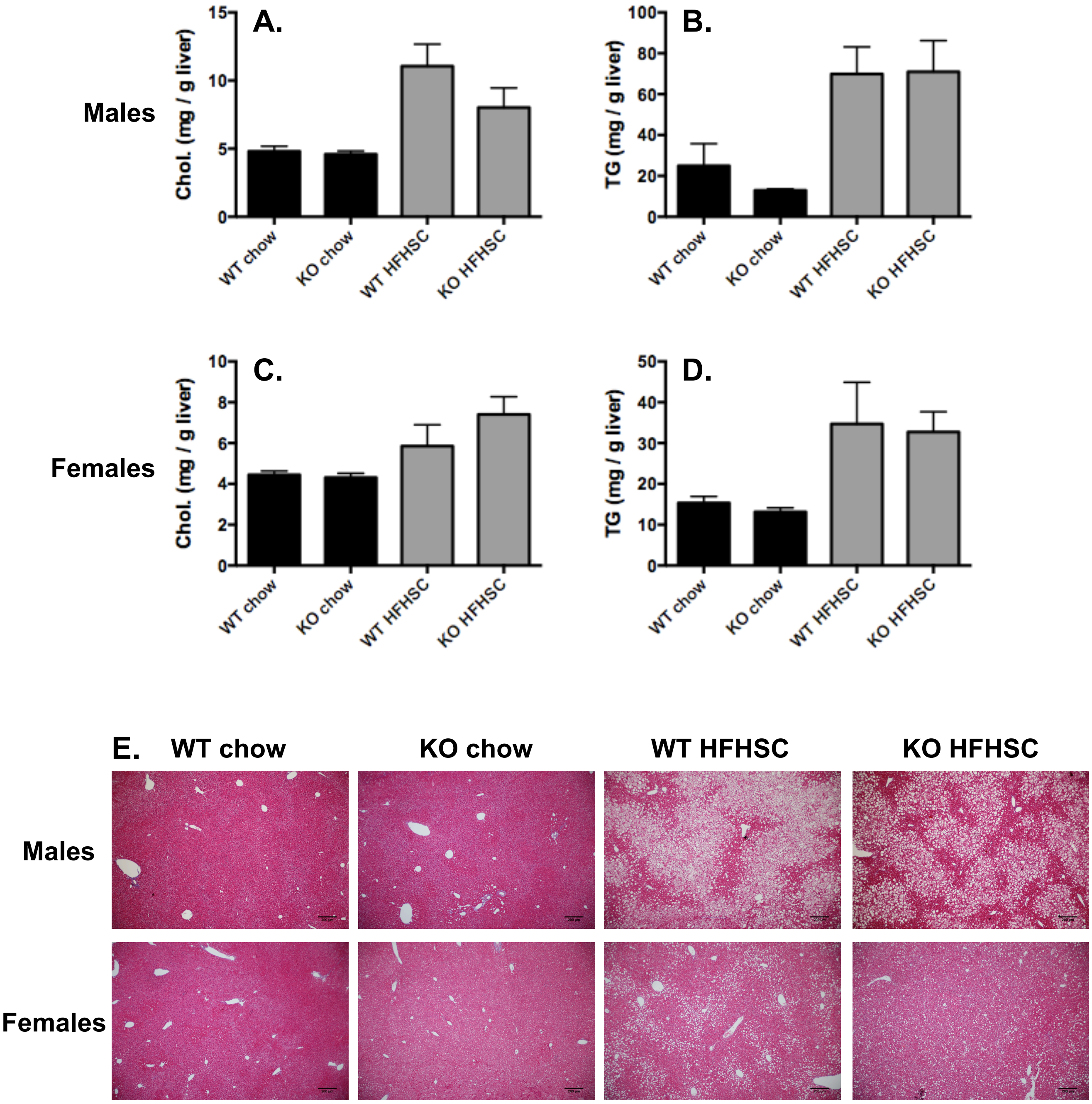

Figure S9. Liver lipids and histology. (A-D) Lipids were extracted from previously frozen liver samples using the Folch method. Cholesterol (Chol.: A,C) and triglycerides (TG: B,D) were quantified. E. Representative images of Masson's trichrome-stained liver sections from male and female, WT (*Saa3*$^{+/+}$) and KO (*Saa3*$^{-/-}$) mice after 16 weeks of chow or HFHSC diet. Scale bar = 200 µm.
